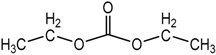# Composite Polymer Electrolytes: Nanoparticles Affect Structure and Properties

**DOI:** 10.3390/polym8110387

**Published:** 2016-11-03

**Authors:** Wei Wang, Paschalis Alexandridis

**Affiliations:** Department of Chemical and Biological Engineering, University at Buffalo, The State University of New York (SUNY), Buffalo, NY 14260-4200, USA; wwang23@buffalo.edu

**Keywords:** solid polymer electrolyte, ionic conductivity, lithium-ion battery, nanoparticle, dielectric properties, transference number

## Abstract

Composite polymer electrolytes (CPEs) can significantly improve the performance in electrochemical devices such as lithium-ion batteries. This review summarizes property/performance relationships in the case where nanoparticles are introduced to polymer electrolytes. It is the aim of this review to provide a knowledge network that elucidates the role of nano-additives in the CPEs. Central to the discussion is the impact on the CPE performance of properties such as crystalline/amorphous structure, dielectric behavior, and interactions within the CPE. The amorphous domains of semi-crystalline polymer facilitate the ion transport, while an enhanced mobility of polymer chains contributes to high ionic conductivity. Dielectric properties reflect the relaxation behavior of polymer chains as an important factor in ion conduction. Further, the dielectric constant (ε) determines the capability of the polymer to dissolve salt. The atom/ion/nanoparticle interactions within CPEs suggest ways to enhance the CPE conductivity by generating more free lithium ions. Certain properties can be improved simultaneously by nanoparticle addition in order to optimize the overall performance of the electrolyte. The effects of nano-additives on thermal and mechanical properties of CPEs are also presented in order to evaluate the electrolyte competence for lithium-ion battery applications.

## 1. Introduction

The emerging notion of fabricating nanoscale functional hybrid materials [1] supported by several novel preparation and modification approaches [2,3] has been employed in various aspects of scientific research [2,4,5]. The application of such hybrids in electrochemical devices such as lithium-ion batteries, fuel cells and supercapacitors is of current interest [2,3,4,6]. 

The lithium-ion battery is one of the most important electrochemical devices for energy storage [7,8,9,10]. A battery is composed of two electrochemically active electrodes [11] separated by an ion conductive, electron insulating electrolyte medium [12]. Rechargeable batteries find widespread usage as portable power sources because of their repeated charge and discharge capability [13]. 

The electrolyte is one of the critical components within the lithium-ion battery [14]. Electrolytes, however, can have several drawbacks. For organic solvent-based electrolytes, problems include intrinsically poor cycling efficiency and flammability [15]. For ionic liquid-based electrolytes, a drawback is their relatively high viscosity which limits the attainable ionic conductivity [16,17,18]. Polymer electrolytes [19,20] have been considered with an aim to overcome such limitations. Despite certain advantages, the archetype polymer electrolyte is based on poly(ethylene oxide) (PEO) with lithium salt dissolved in it [21]. The semi-crystalline structure of PEO presents inherent problems as a polymer matrix for Li^+^: (1) not sufficiently high ionic conductivity, especially at ambient temperature; (2) insufficient mechanical strength; (3) dendrite growth at the interface between electrolyte and electrode which might cause internal short circuit [8].

In order to overcome these limitations, several avenues have been explored. One promising line of investigation involves the introduction of nano-sized additives [22,23] in order to minimize the concentration of PEO crystalline domains without harming the PEO flexibility and mechanical stability over a wide temperature range. Such additives encompass inert oxide ceramics, molecular sieves and zeolites, rare-earth oxide ceramics, solid super acids, ferroelectric materials and carbon [24]. The effects of the inorganic additives have been analyzed in terms of Lewis acid-base interactions [22] between the surface groups of the additives and active sites on the polymer chains. Different effects can either impair or enhance the ionic conductivity. For example, inorganic additives change the interfacial resistance between electrolyte and electrode [25]. When these effects are combined, the enhancing effect may be dominant or otherwise the impairing effect may be stronger. This might explain the different results of similar systems with nanoparticle addition. 

Despite many studies concerning nano-sized additives for polymer electrolytes, several issues remain unresolved [24,26]. The purpose of this review is to provide a knowledge network that helps to assess the role of nano-additives in the CPEs, and to guide the readers toward directions that merit further research effort. Although several types of polymers [27] can serve as matrices to dissolve lithium salts, including polyacrylonitrile (PAN), poly(vinylidene difluoride) (PVdF), poly(methyl methacrylate) (PMMA) [28], and poly(propylene oxide) (PPO) [29], in this review we focus on conducting matrices based on PEO. And while several conducting cations have been considered, e.g., Ag^+^, Na^+^ [30,31,32,33,34], this review focuses on Li^+^ for applications in lithium-ion batteries.

**Figure 1:**
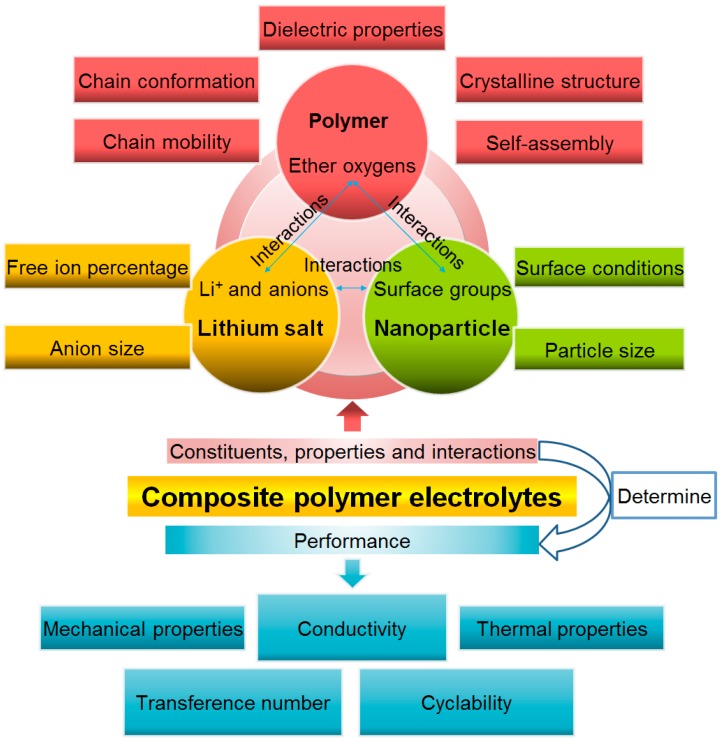
Knowledge network for the study of composite polymer electrolytes.

This review is founded on the premise that the CPE performance is determined by factors such as structure and interactions between atoms/ions/nanoparticles within the system, and dielectric properties as shown in Figure 1. These factors, in connection to each constituent of the CPE, are addressed in Chapters 3 and 4, in combination with the corresponding conductivity change, in order to elucidate the influence of these factors on the CPE performance. 

This is a comprehensive and up-to-date review focusing on polyether-based composite polymer electrolytes for lithium-ion batteries that addresses property/performance relationships. Previous reviews have either focused on different types of additives [22,24] or different polymer matrices [20,26], or on other charge carriers, like Na^+^ [23], or on other electrochemical devices, like fuel cells [2,35], or addressed the homogeneity [23] of the systems, or limited the discussion on the structure and morphology of the polymer electrolytes [1,20,36]. 

Ion transport models for polymer electrolytes are introduced in Chapter 2. Crystallinity, chain conformation, chain mobility and interactions between atoms/ions/nanoparticles within the CPE system are discussed in Chapter 3. Chapter 4 addresses dielectric properties for an analysis of the polymer relaxation behavior and an understanding of ion conduction behavior in CPEs. The factors discussed in Chapter 3 and 4 critically determine the performance of the electrolytes. Thermal and mechanical properties are reviewed in Chapters 5 and 6 in the context of electrolyte performance in practical applications. Throughout this review we discuss how properties and interactions between individual components within the CPEs affect the electrolyte performance.

## 2. Ion Transport in Binary and Composite Polymer Electrolytes

For the application of polymer electrolytes in lithium-ion batteries, the most critical requirement is the ionic conductivity, the conductivity discussed in this review refers to ionic conductivity unless indicated otherwise. Thus, we start by introducing ion transport models for polymer electrolytes. These models are commonly used to describe the ion conduction behavior in binary polymer electrolytes (i.e., systems consisting of polymer and lithium salt) and are also employed for ternary polymer electrolytes (systems of polymer, lithium salt, and additive).

The Vogel-Tamman-Fulcher (VTF), Willams-Landel-Ferry (WLF), and Arrhenius equations [37,38] are broadly utilized to describe the ion conduction behavior of CPEs. The VTF equation is mostly applied for amorphous ionic conductivity, while the Arrhenius equation is often applied for crystalline ionic conductivity [39,40]. For high molecular weight polymer electrolytes, amorphous ion conduction is prevalent, thus we focus here on the VTF equation. 

The VTF equation was developed to describe the viscosity of supercooled liquids [41]:

(1) $$\eta =C\exp[B/(T-T_{0})]$$

where the pre-exponent coefficient *C* ∝ *T*^1/2^, *B* is related to the activation energy, and *T*_0_ is a reference temperature. When Equation (1) is combined with the Stokes-Einstein equation (Equation (2)) and the Nernst-Einstein equation (Equation (3)), Equation (4) results:

(2) $$\sigma =\frac{nq^{2}}{kT}D$$

(3) $$D=\frac{kT}{6\pi r_{i}\eta }$$

(4) $$\sigma =\sigma_{0}\exp[-B/(T-T_{0})]$$

In Equation (2), *n* is the carrier concentration, *q* is the carrier charge, *D* is the carrier diffusion coefficient, and *k* is Boltzmann’s constant. In Equation (3), *r*_i_ is a diffusion radius. In Equation (4), *T*_0_ is a reference temperature roughly 50 K below the glass transition temperature [42]; the pre-exponent coefficient σ_0_ ∝ *T*^−1/2^.

Although the VTF equation was initially an empirical expression, theories have been subsequently employed to strengthen its validity. The free volume [43] and the “Corrected Free-Volume” [44] theories both lead to an expression similar to the VTF form:

(5) $$\sigma =AT^{-1/2}\exp[-E_{a}/(T-T_{0})]$$

Besides the free-volume theory that was put forward from a macroscopic, thermodynamic point of view, from a microscopic view, the percolation [45,46] and the dynamic bond percolation [47] models can also be applied for the case of solid polymer electrolytes where small particles (alkali ions) diffuse within the dynamic motion of the medium (polymer). 

**Figure 2:**
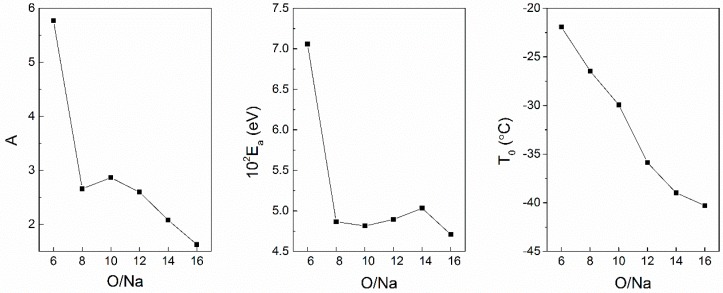
Variation of three parameters: pre-exponential factor (*A*), activation energy (*E*_a_), reference temperature (*T*_0_) within the Vogel-Tamman-Fulcher (VTF) equation for poly(propylene oxide) (PPO)-sodium triflate system at different salt concentrations expressed in the *X*-axis as O/Na ratio. Data from Armand et al. [20].

Equation (5) has been applied to the ion conduction behavior in polymer electrolytes [48]. For example, parameters of the VTF equation are plotted in Figure 2 versus salt concentration for a PPO-sodium triflate (sodium trifluoromethanesulfonate, CF_3_SO_3_Na) system [20]. In this system, the pre-exponential factor (A) (frequency factor) in Equation (5) increased with increasing salt concentration. The trend is similar for the activation energy *E*_a_, which showed a turning point at molar ratio *n* = O:Na^+^ = 8. This *E*_a_ trend informs the change of energy barriers for ion transport in the polymer electrolytes. The glass transition temperature *T*_0_ (pure polymer *T*_0,(n = ∞)_) decreased monotonically with dilution. An analysis of the dependence of these parameters on experimental variables provides insights into the ionic conductivity change of polymer electrolytes.

The Arrhenius equation is also commonly used to describe the ionic conductivity in crystalline polymers [39,40]:

(6) $$\sigma =\sigma_{0}\exp(-E_{a}/kT)$$

Because of the easiness to obtain the activation energy (*E*_a_) and pre-exponential factor (σ_0_), the Arrhenius equation is also widely used to express ionic conductivity for amorphous conduction. For the case of fast ionic conductors, the relation between σ_0_ and *E*_a_ in the Arrhenius equation can be described by the semi-empirical Meyer-Neldel (MN) rule [49]:

(7) $$\log\sigma_{0}=\alpha E_{a}+\beta =\frac{E_{a}}{kT_{D}}+\ln K\omega_{0}$$

where *T*_D_ is a characteristic temperature, *k* is the concentration term and ω_0_ is the ion attempt frequency (hopping rate between adjacent adsorption sites [50,51]) related to σ_0_ [23]. The Meyer-Neldel (MN) rule is also applicable for mixed-phase (inhomogeneous polymer and additive that are not in a common solvent) and blend-based (homogeneous solution of two components in a common solvent) polymer electrolytes [52,53].

In closing, the VTF model for binary solid polymer electrolytes captures data for composite polymer electrolyte systems reasonably well, which suggests that the conduction mechanism and associated model do not change upon the incorporation of nano-additives.

## 3. Nanoparticle Additives Affect the Polymer Electrolyte Structure

A key property of a polymer electrolyte is the conductivity [20,23,24,28,54]. The performance of the polymer electrolyte is greatly determined by the polymer structure as it constitutes the matrix for ion transport. The mobility of polymer chains [55] and the interactions of lithium ions [56,57] within the polymer matrix greatly determine the conduction behavior of polymer electrolytes. The second factor will be discussed in detail in Section 3.5. Regarding the first factor, for high molecular weight polymer-based electrolytes, the amorphous polymer domains [58,59] account primarily for the ion transport whereas the crystalline counterparts hinder ion movement. (Note that ion transport in crystalline domains has been reported in low molecular weight PEO [60,61], but the discussion in this review focuses on amorphous conduction.) The mobility of the polymer chains also affects ion conduction. In the first three sub-sections of Chapter 3 we review the effects of nanoparticles on the polymer chain (1) structure; (2) conformation; and (3) segmental movements.

### 3.1. Effect of Nanoparticles on Polymer Crystallinity

**Figure 3:**
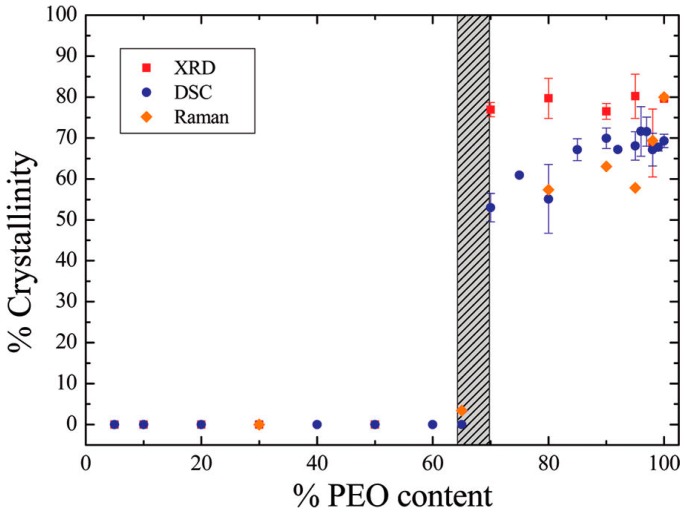
Degree of crystallinity of poly(ethylene oxide) (PEO)/Na^+^–MMT hybrids at 90 °C, plotted as a function of PEO weight %, obtained from the analysis of X-ray diffraction (XRD) (**filled squares**), differential scanning calorimetry (DSC) (**filled circles**), and Raman spectroscopy (**filled diamonds**) data. Reproduced with permission from Chrissopoulou et al. [62]. Copyright 2011, American Chemical Society.

Given that the structure of polymer electrolytes (e.g., amorphous or crystalline) plays a prominent role in facilitating ion transport, the effect of nanoparticles on the fraction of amorphous domains in CPEs becomes critical. We start by discussing the crystallinity of polymer-nanoparticle systems in the absence of lithium salt. The crystallinity of PEO (molecular weight = 100,000 g/mol, polydispersity index *M*_w_/*M*_n_ = 2.4)-sodium montmorillonite (cation exchange capacity 92.6 mmol/100 g, particle size not reported) hybrids [62] (without lithium salt) is presented in Figure 3. When PEO < 70 wt %, the crystalline peaks of X-ray diffractograms all disappear, which indicates that the hybrid system is almost amorphous. However, when PEO > 70 wt %, the PEO crystallinity seems rather constant and independent of the PEO content. The results were explained on the basis of two opposing effects: [62] crystallization enhancement by the addition of inorganic fillers, and crystallization hindrance by the coordination of alkali cations with polymer chains.

When nanoparticles are added into a binary system of polymer with lithium salt, a different behavior is observed. For example, CdO nanoparticles (2.5 nm diameter) were added to PEO (400,000 g/mol)–LiI electrolyte at a fixed molar ratio of EO/Li^+^ = 12 (the CdO content in the system was 0.05–0.20 wt %) [63]. Because of the LiI in the system, the crystallinity of the polymer was already lowered compared to the neat polymer (Table 1) because of the coordination of alkali cations with polymer chains. The introduction of CdO at first decreased the crystallinity [63]. A minimum value of crystallinity was achieved at 0.10 wt % CdO. Further addition of CdO led to a crystallinity increase. This result is comparable with the following example, in which the EO/Li^+^ ratio was set as a variable [64]. In PEO (300,000 g/mol)–LiClO_4_, a fixed amount of 10 wt % SiO_2_ (diameter = 10 nm, Specific Surface Area = 956 m^2^/g) was introduced, which reduced crystallinity from 48.5% for the SiO_2_-free sample to 44.3% in the temperature range −75–100 °C. As the EO/Li^+^ molar ratio increased to 12, the polymer turned all amorphous. 

The effect of CdO nanoparticles on crystallinity has also been evidenced in the Field Emission Scanning Electron Microscope (FE-SEM) images of spherulites in PEO-LiI-CdO CPEs. The average diameter of spherulites for the additive-free PEO-LiI electrolytes is around 120 μm. When CdO was added into PEO-LiI electrolytes, the PEO spherulites increased in number and decreased in average size to about 50 μm. This indicates that CdO addition is beneficial for the reduction of the PEO degree of crystallinity [65,66]. As for the reason, CdO serves as a nucleation center for spherulite initiation and because the number the sperulites increases, the size of an average spherulite is reduced. However, it was noticed that as the CdO concentration increased, the spherulite size would not change appreciably which might be explained in that not all CdO added could effectively act as nucleation sites. Aside from this, the variation of the amorphous phase content was dramatic. 

The importance of polymer crystallinity in the study of CPEs cannot be overrated. Crystallinity is affected by temperature, amount of lithium salt and nanoparticle changes [26,62,63,64,67,68]. Crystallinity underlies the performance of CPEs such as conductivity and mechanical strength. 

### 3.2. Effect of Nanoparticles on Polymer Chain Conformation

**Figure 4:**
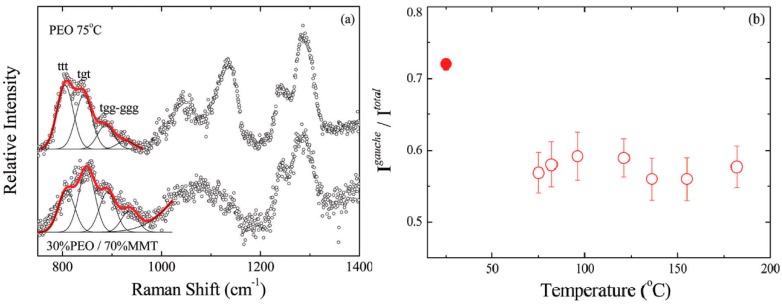
(**a**) Raman spectra at 75 °C of neat PEO and a 30 wt % PEO/70 wt % sodium-activated montmorillonite (Na^+^-MMT) nanocomposite at ambient temperature and their fit with four Gaussians in the spectral region of CH_2_ rocking vibrations; (**b**) Ratio of integrated intensity of the bands that correspond to gauche conformation to the total integrated intensity in the spectral region of CH_2_ rocking vibration. PEO (**open circles**); 30 wt % PEO/Na^+^–MMT (**filled circles**). Reproduced with permission from Chrissopoulou et al. [62]. Copyright 2011, American Chemical Society.

The polymer chain conformation influences the dynamics of the lithium ions coordinated with the polymer backbones [69] because Li^+^ ions exhibit different binding energies with the different conformers [70]. The polymer chain conformation can change due to nanoparticle addition as shown by Raman [62] and FTIR spectroscopy [67,71,72]. In the 700–1600 cm^−1^ Raman spectra of a PEO-sodium montmorillonite system, there are four characteristic regions corresponding to four types of vibration bands: CH_2_ rocking vibrations, C–O–C stretching vibrations, CH_2_ twisting vibrations and CH_2_ bending vibrations [62]. As temperature changed or the PEO content of the system varied, the intensity in the CH_2_ rocking vibrations region (750–970 cm^−1^) changed, suggesting conformation changes of polymer chains [62]. As shown in Figure 4b, the gauche conformation in neat polymer accounts for 55%–60% in the temperature range 75–180 °C. When sodium-activated montmorillonite (Na^+^-MMT) (cation exchange capacity 92.6 mmol/100 g) was added, the gauche percentage increased to 72% at ambient temperature [62]. Because salt may form transient crosslinks with polymer chains [36], the effect of salt on the polymer chain conformation merits further investigation in the presence and in the absence of nano-sized additives.

Conformational perturbations would be expected to affect the dynamics of Li^+^ ion coordination to the PEO backbone and ion mobility [69], which lead to conductivity changes. As for a quantitative analysis of polymer chain conformation effects on polymer electrolyte conductivity, molecular dynamic simulation studies are available [73,74] but experimental evidence appears difficult to obtain. This is because the conformation change is brought by certain experimental conditions, e.g., temperature variation or nanoparticle addition, which possibly exert a more significant effect on conductivity. Thus, it is hard to isolate the contribution of conformation change to the ionic conductivity.

**Figure 5:**
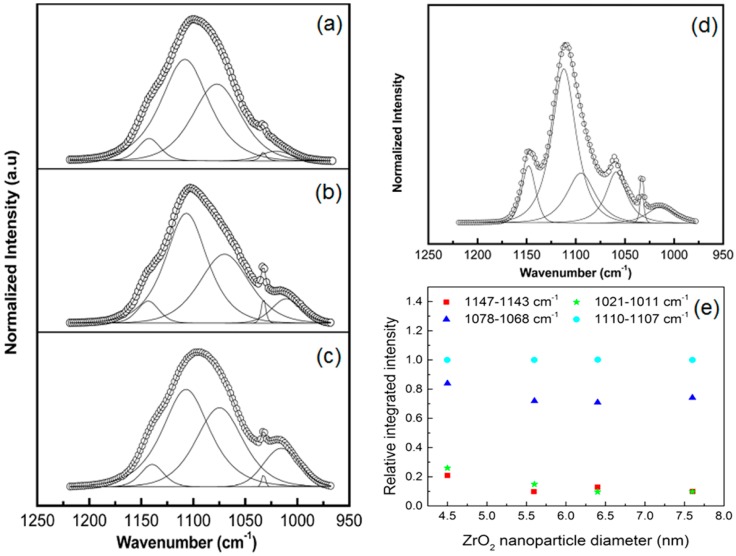
Deconvolution of Fourier Transform Infrared (FTIR) spectra for C-O-C band in (**a**) PEO-NaClO_4_ (EO:Na^+^ = 25); (**b**) solid composite polymer electrolyte PEO-NaClO_4_ (EO:Na^+^ = 25) with ZrO_2_ nanoparticles (d = 4.5 nm); (**c**) solid composite polymer electrolyte PEO-NaClO_4_ (EO:Na^+^ = 25) with ZrO_2_ nanoparticles (d = 7.6 nm); (**d**) neat PEO in the region of 1250–950 cm^−1^; (**e**) Variation of relative integrated intensity with particle size of ZrO_2_ for four peaks in the region 1147–1011 cm^−1^. Data from Dey et al. [67].

In addition to Raman, Fourier Transform Infrared (FTIR) spectroscopy can also be employed to obtain information on polymer chain conformation in CPEs [67,72]. In the PEO–NaClO_4_ (EO:Na^+^ = 25) system, results have been presented with a deconvolution of C–O–C within the wavelength range of 950–1250 cm^−1^ [67]. Figure 5a–c shows FTIR spectra for PEO–NaClO_4_ (EO:Na^+^ = 25) and for the same system but with 4.5 and 7.6 nm ZrO_2_ (5 wt %) addition. When sodium salt and nanoparticles were added, the main absorption band of PEO in the polymer-salt complex shifted from 1112 cm^−1^ to a lower value of 1108 cm^−1^, and its full width at half maximum (FWHM) broadened to 53 cm^−1^. This suggested the coordination of ions with ether oxygens. The peak at the 1059 cm^−1^ position associated with the crystalline structure in pristine PEO almost disappeared, indicating a decrease of crystallinity. Figure 5d shows the FTIR spectra of C–O–C vibrational mode of pristine PEO. Within the fitted spectral region of 1200–1000 cm^−1^ of the FTIR spectrum, there are six peaks in the positions of 1014, 1033, 1059, 1094, 1112, and 1148 cm^−1^, which correspond to C–O–C symmetric and asymmetric modes. The maximum of the peak is at 1112 cm^−1^ with FWHM = 29 cm^−1^. The changes of the spectra revealed that different interactions within the system caused the deformation on the C–O–C bond angle and changes in the stretching module, which led to changes of the integrated area ratio as shown in Figure 5e.

Although its effect is not as strong as crystallinity, the chain conformation responds to various conditions, e.g., particle size and temperature change. This conformation analysis contributes to a better understanding of composite polymer electrolytes.

### 3.3. Effect of Nanoparticles on Polymer Chain Segmental Movement

Following the discussion about “static” chain conformation in Section 3.2, we address here the polymer segmental motion as a dynamic counterpart with an aim to understand how the polymer matrix facilitates ion movement. In order to study the effect of nanoparticles on polymer chain segmental mobility, nuclear magnetic resonance (NMR) has been employed to analyze two characteristic temperatures, the glass transition temperature, *T*_g_^NMR^, and the temperature which corresponds to the maximum spin-lattice relaxation rate, *T*_max_ [75]. Additionally, the line widths (Δ*ν*) and the spin-lattice relaxation times (*T*_1_) of the ^1^H, ^13^C, and ^7^Li nuclei as affected by temperature have been employed to study the mobility of the ions and the polymer chains [76,77]. 

Different samples of PEO/silica with lithium salts have been prepared and denoted as [*X*]_n_[*Y*]-*Z*, where *X* is the polymer weight percentage, *n* is the average number of repeating units in polymer chains, *Y* is the ratio of ether oxygens to Li^+^, *Z* is either I or II, standing for non-bonded (i.e., each silicate group only bonded to each other through oxygen bridge in the silica phase) or bonded (silicate group bonded to polymer chains by covalent bonds) complex structure, respectively [76]. As temperature changes, the ^7^Li NMR line width (FWHM, Δ*ν*) would undergo narrowing, which is commonly associated with chain motion increase. The corresponding temperature is close to the *T*_g_ value obtained from DSC and thus is denoted as *T*_g_^NMR^. Another temperature that corresponds to the maximum spin-lattice relaxation rate is denoted as *T*_max_, where transverse local field fluctuations suffice to maximize the rate of spin-lattice relaxation at the Larmor frequency [76]. These two parameters, as listed in Table 2, have been employed to analyze polymer chain mobility. 

The results from Table 2 can be summarized as follows. For non-bonded complexes of type I: (1) in Series 1, decreased *T*_g_^NMR^ and *T*_max_ when *X* > 80 indicate a reduced mobility of the polymer domains adjacent to silica clusters; (2) in Series 2, longer chain length increased chain hindrance and thus increased *T*_g_^NMR^ and *T*_max_ [78]; (3) in Series 3, upon addition of silica, *T*_g_^NMR^ and *T*_max_ remain approximately constant for samples with different amount of lithium salt, the result being different from those systems without silica [79]. Thus, it can be concluded that polymer-silica interactions weakened the effect of lithium salt on polymer chain mobility. For bonded complexes of type II: in Series 4, salt addition led to increased *T*_g_^NMR^ and *T*_max_ as expected, since lithium ions form transient crosslinks with polymer chains that reduce mobility.

**Figure 6:**
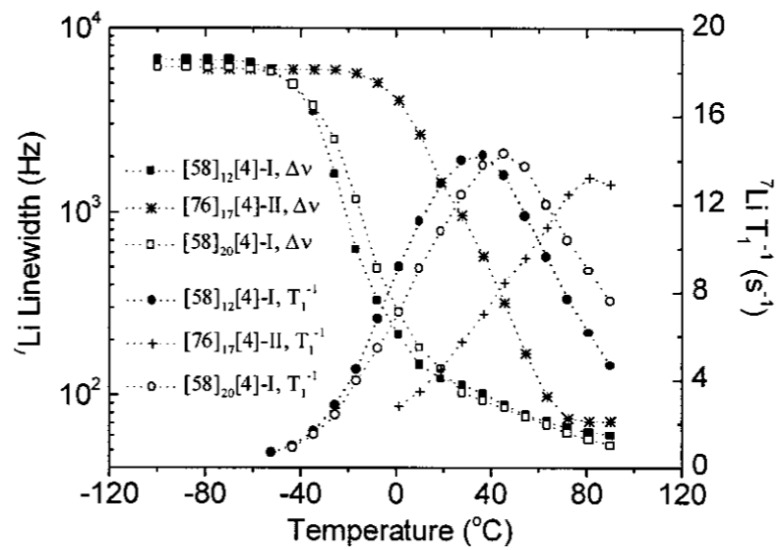
Temperature dependence of the ^7^Li spin-lattice relaxation rate and line width without ^1^H decoupling for the nanocomposites [58]_12_[4]-I, [58]_20_[4]-I, and [76]_17_[4]-II in Table 2. Reproduced with permission from Mello et al. [76]. Copyright 2000, American Chemical Society.

A typical example that demonstrates the effectiveness of NMR in providing insights on the conductivity change follows. Figure 6 is a plot of ^7^Li line width without ^1^H decoupling (Δ*ν*) and ^7^Li spin-lattice relaxation rate (*T*^−1^) versus temperature for three different nanocomposites: [58]_12_[4]-I, [58]_20_[4]-I, and [76]_17_[4]-II. We can see an obvious *T*_g_^NMR^ and *T*_max_ increase of bonded sample of type II as shown by a shift of the Δν and *T*^−1^ plot to the higher temperature, in contrast to non-bonded sample of type I. This suggests that covalent bonding between silica particle and the PEO chains hinders the polymer chain motion and also the Li^+^ mobility, accompanied by an ionic conductivity drop [76]. This result supports the general conclusion that polymer electrolytes containing nanoparticles (for the case of non-bonded samples) exhibit improved conductivity. The chemical bonding between nanoparticles and polymer chain does not always increase the CPE ionic conductivity. 

### 3.4. Effect of Nanoparticles on Polymer Self-Assembly and Anisotropic Conductivity

We are interested in polymer self-assembly in composite polymer electrolytes because (i) nanoparticles in the system affect the micro-phase separation of the polymer matrix [80,81,82] and (ii) polymer self-assembly (microphase separation or phase transition) creates different environments for ion conduction and both ionic and electric conductivities vary accordingly. We address this topic here briefly; however, extensive efforts have been devoted to block copolymer thermodynamics [5,83,84,85] in the melt state [86,87,88,89,90] and in solution [90,91,92,93]. The introduction of nanoparticles into block copolymer electrolyte systems typically leads to an increase of the segregation strength (χN) to generate different microphase separated morphologies [94,95,96]. Block copolymers can change from the disordered state to form spherical, cylindrical, lamellar, gyroid or other morphologies depending on factors such as the block ratio, solvent medium, and hydrogen bonding of the ligands [97] with polymer chains [98,99,100]. Here we highlight (1) the effect of nanoparticles on the segregation of block copolymer-salt electrolytes [101] and (2) how self-assembly can affect the performance of CPEs.

**Figure 7:**
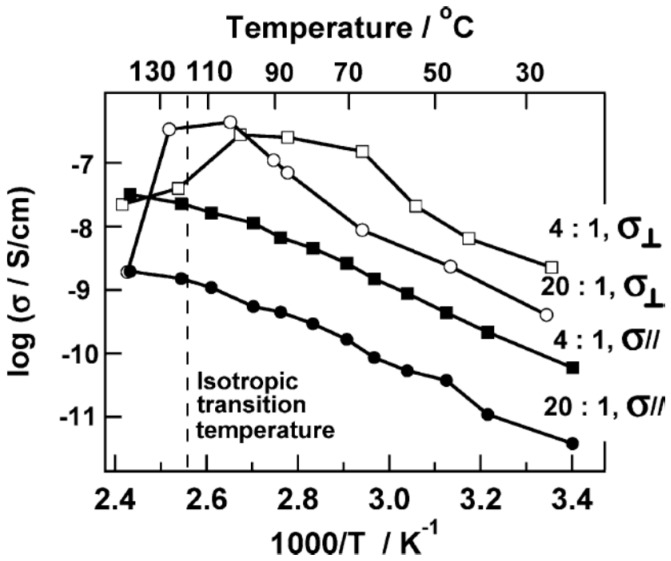
Anisotropic ion conductivity of PEO_114_-*b*-PMA(Az)_47_–LiCF_3_SO_3_ complexes plotted as a function of temperature for EO:Li^+^ ratios of 20:1 and 4:1, in the micro-domains perpendicular (**open symbols**) and parallel (**solid symbols**) to the substrate. Reproduced with permission from Li et al. [102]. Copyright 2007, American Chemical Society.

Anisotropic ion transport behavior was reported in microphase-segregated electrolyte membranes of (PEO_114_)-*b*-poly(methyl acrylate) with azobenzene mesogen [PMA(Az)_47_]–LiCF_3_SO_3_ as shown in Figure 7 [102]. Upon comparing the perpendicular conductivity (where cylindrical domains of PEO are perpendicular to the electrodes, thus current moves along the cylindrical domains of PEO) with the parallel conductivity (cylindrical domains of PEO are parallel to electrodes, thus current transverses the cylindrical domains of PEO), it was found that the perpendicular conductivity σ_⊥_ facilitated by a perpendicularly aligned PEO cylindrical array was always higher than the parallel conductivity σ_∥_. The parallel conductivity σ_∥_ exhibited a monotonic increase as the temperature increased for samples with EO:Li^+^ = 20:1 and 4:1. The perpendicular σ_⊥_ attained a maximum at around 377 K, followed by a drop [102]. 

**Figure 8:**
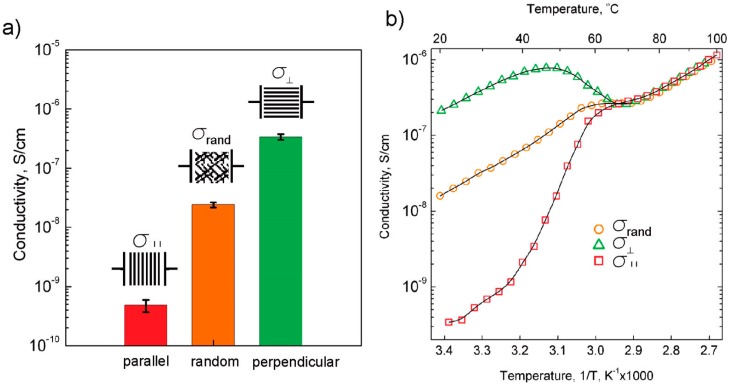
(**a**) Average room temperature conductivity of poly(ethylene oxide-*b*-6-(4′-cyanobiphenyl-4-yloxy)-hexyl methacrylate) PEO-b-PMA/CB (10.4 kg/mol, PDI = 1.15, *f*_PEO_ = 0.23, EO:Li^+^ = 120:1) aligned in 5 T magnetic field in two orthogonal directions; the nonaligned material conductivity is shown for comparison; (**b**) Temperature dependence of conductivities shown in an Arrhenius-type plot (*x*-axis reversed). Reproduced with permission from Majewski et al. [103]. Copyright 2010, American Chemical Society.

Similarly, for a poly(ethylene oxide)-*b*-6-(4′-cyanobiphenyl-4-yloxy)-hexyl methacrylate) (PEO-b-PMA/CB) polymer matrix doped with LiClO_4_ (EO:Li^+^ = 120:1), a magnetic field was used to induce micro-domains of highly aligned hexagonally packed cylinders [103]. The conductivity of parallel PEO cylinders (σ_∥_) was lower than that of randomly oriented sample (σ_rand_) by more than one order of magnitude at room temperature. The perpendicular PEO cylinder conductivity (σ_⊥_) was higher than that of a randomly oriented sample by one order of magnitude as shown in Figure 8a. In Figure 8b, σ_⊥_, σ_∥_ and σ_rand_ overlapped at high temperatures until *T*_ODT_, i.e., the temperature where order-disorder transition (ODT) occurred. When *T* < *T*_ODT_, σ_⊥_, σ_∥_ and σ_rand_ responded differently with temperature increase. The origin of distinct behaviors of conductivity in aligned samples below *T*_ODT_ was still unclear [103]. 

Further research can examine the effect of nanoparticles on other micro-phase separated morphologies, e.g., aligned lamellae or bicontinuous structures. The number of nanoparticles that could be accommodated in the polymer matrix remains unresolved. It is postulated that a stronger interaction between ligand and the polymer chains allows a higher incorporation level and the ordered polymer structure can be maintained at a higher content of additive [97]. In closing, the effect of nanoparticles on block copolymer segregation and the anisotropic conductivity of block copolymer electrolytes is a topic of continuous interest [101,104].

As mentioned at the beginning of Chapter 3, atom/ion/nanoparticle interactions within the polymer matrix greatly affect the conduction behavior of polymer electrolytes [56,57]. After the discussion about the polymer matrix structure, polymer chain conformation and segmental movement, and block copolymer self-assembly, we now proceed to discuss the interactions between atoms, ions, and nanoparticles inside the polymer matrix. Thus, in what follows, we address (1) interactions within CPEs in Section 3.5; (2) lithium salt dissociation versus ion pairing and ion aggregates in Section 3.6; and (3) cation transference number *t*^+^ as the contribution of lithium ion to the charge transport in Section 3.7. 

### 3.5. Interaction of Nanoparticles with Polymer Chains

Ion transport can be either hindered or facilitated by interacting polymer chains and nanoparticles. The introduction of nanoparticles increased conductivity as reported by many authors [22,23,24,26]. Oxygen vacancies at the surface of nanoparticles (e.g., SnO_2_) are thought to act as Lewis acids to coordinate with either ether oxygens of polymer chains or anions of lithium salt as Lewis bases. Based on this understanding, we need to obtain information about the “oxygen vacancy percentage” on the surface of nanoparticles. Such information can be obtained from X-ray photoelectron spectroscopy (XPS). According to XPS spectra of oxygen of SnO_2_ nanoparticle for the system PEO (600,000 g/mol)–LiClO_4_ (EO:Li^+^ = 8)–SnO_2_ (*d* = 3–4 nm, weight ratio of SnO_2_:PEO = 0.05, 0.10, 0.15, 0.20) [105], two types of oxygens are seen: (1) structural O, from Sn–O bond and (2) absorbed O, from O_2_ and CO_2_ in the atmosphere. By peak deconvolution, the percentage of structural O in the systems and its ratio to Sn can be estimated (Table 3) [105]. The theoretical Sn/O ratio is 0.5 for SnO_2_. Assuming the Sn atom amount in each sample is 100, the results in Table 3 show much larger Sn/O value. This indicates many oxygen vacancies present on the surface of SnO_2_ nanoparticles [105].

**Figure 9:**
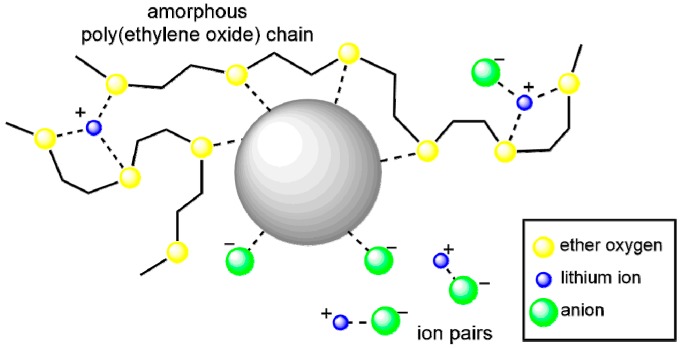
Schematic of Lewis acid-base interactions between a PEO–LiClO_4_ electrolyte host and a nanoparticle guest.

Figure 9 illustrates the interaction of nanoparticles within polymer electrolytes. The oxygen vacancies on the nanoparticle surface (as Lewis acid) coordinate in two ways: (1) with ether oxygens of the polymer chain, to hinder PEO crystallization and produce higher amorphous fraction; and (2) with oxygens from anions of lithium salt (LiClO_4_) [105,106,107,108], to reduce the ion pair (Li^+^–ClO_4_^−^) and release more free Li^+^ ions. Both effects contribute to enhance the conductivity of the CPEs. After the “oxygen vacancy percentage” on the surface of nanoparticles has been obtained, this notion can be combined with the topic of “free ion percentage” in Section 3.6 for more detailed interaction analysis.

In order to improve the details of the scheme shown in Figure 9, the distance between lithium ions and carbons of polymer chains (Li^+^–Carbon distance) and the coordination number (the number of oxygens coordinated with one lithium ion) are required. Rotational Echo Double Resonance (REDOR) has been employed to measure dipolar and quadrupolar coupling values between NMR active nuclei to determine intermolecular distances in the solid state [109,110]. For example, ^7^Li–^13^C quadrupolar signals indicated that Li–C distance = 3.14 Å [111]. This result compared favorably with that of 2.23–4.27 Å obtained from neutron powder diffraction [112].

### 3.6. Single Ions versus Ion Pairs and Ion Aggregates within Composite Polymer Electrolytes

After the discussion of interactions between nanoparticles and binary polymer electrolytes, we now focus on the lithium salt. In the polymer electrolyte, lithium salt ions exist in the forms of (1) single ions; (2) ion pairs and (3) ion aggregates [56,57] but only single ions contribute to the ionic conductivity. Thus it is desirable to enhance the ion dissociation in the polymer matrix in order to improve the CPE performance. The percentage of free lithium ions can be studied by FTIR, Raman and NMR [67,110,113]. 

**Figure 10:**
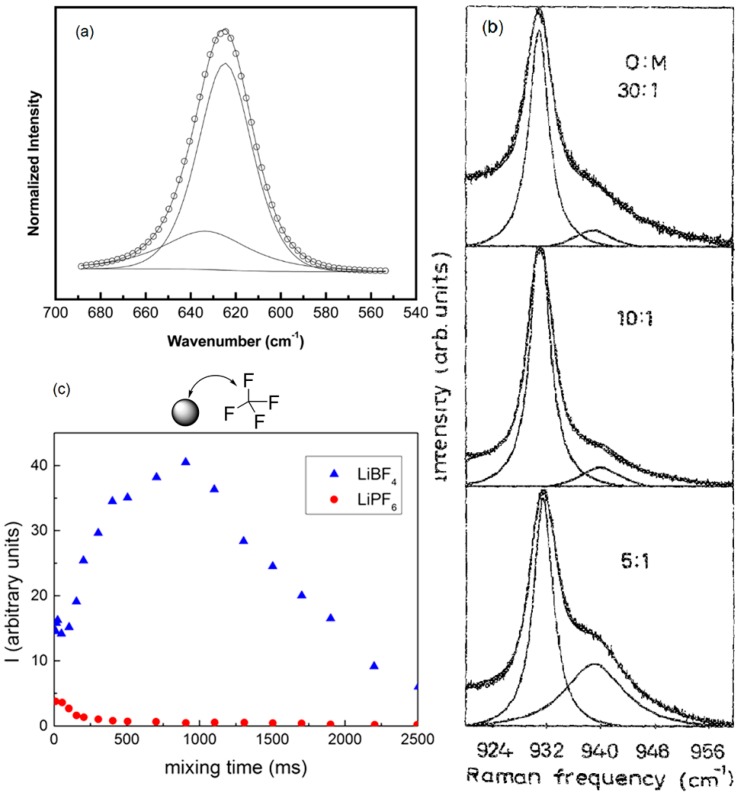
(**a**) Peak fitting of FTIR spectra for ν(ClO_4_^−^) in PEO electrolytes containing ZrO_2_ of 6.4 nm in diameter. Reproduced with permission from Dey et al. [67]. Copyright 2011, American Institute of Physics; (**b**) ClO_4_^−^ symmetric stretching mode for different O:M (ratio of ether oxygen to metal ion) concentrations of LiClO_4_-complexed poly(propylene glycol). The peak positions at 930 and 940 cm^−1^ correspond to free ion and ion pairs, respectively. The experimental profiles have been fitted to a convolution curve consisting of two Lorentzian components. Reproduced with permission from Schantz et al. [113]. Copyright 1988, American Institute of Physics; (**c**) Heteronuclear Overhauser Spectroscopy: Evolution of the ^19^F–^7^Li Nuclear Overhauser Effect (NOE) signal with the mixing time for two lithium salts. LiBF_4_ forms strong ion pairs while LiPF_6_ is dissociated. Data from Judeinstein et al. [110].

The addition of nanoparticles into polymer electrolytes can enhance the salt dissociation to produce more free cations for conductivity. The percentage of free anions in PEO (1,000,000 g/mol)–NaClO_4_ (EO:Na^+^ = 25) system was 68% [67]. When 5 wt % of ZrO_2_ (*d* = 4.5 nm) was added, this percentage increased to 81% as obtained from the FTIR peak integration ratio. However, larger ZrO_2_ particle size (*d* = 7.6 nm) caused a slight decrease in the percentage of free anions. This was attributed to stronger Lewis acid-base interaction resulting from comparatively larger surface area of 4.5 nm ZrO_2_, which produce more free ClO_4_^−^ ions in the solid polymer electrolyte system [67]. Fitted Gaussian-Lorentzian peak of the ClO_4_^−^ of FTIR pattern [67] is shown in Figure 10a: the ClO_4_^−^ band has been well fitted into two peaks centered at wavenumber of 624 and 633 cm^−1^ corresponding to free anions and contact ion pairs, respectively [114,115]. 

The free ion percentage is affected not only by nanoparticle addition but also the lithium salt concentration. Figure 10b shows Raman spectra of the anion symmetric stretching mode for ClO_4_^−^ for different salt concentration in the system poly(propylene glycol) (4000 g/mol)–LiClO_4_ (O:Li = 30, 10, 8, 5) [113]. The fraction of ion pairs increased with salt amount for all concentrations. 

The effect of different anions on lithium salt dissociation has been studied by NMR which also provides estimates about ion pairing and contact between certain solvent moieties by homo- or hetero-Nuclear Overhauser Effect measurements (abbreviated as NOESY or HOESY) [110]. The HOESY methodology can be used, for example, to study the ion-pairing between LiBF_4_ and LiPF_6_ in PEO melt. Figure 10c presents a comparison of the NOE effect for both LiBF_4_ and LiPF_6_ in block oligomer C_5_H_11_NHCONH(CH_2_CH_2_O)_11_NHCONHC_5_H_11_. The bell-shaped profile of LiBF_4_ salt indicated strong ion-pair formation in the 100–500 ms timescale; while the quasi null Nuclear Overhauser Effect (NOE) effect for LiPF_6_ ions revealed the much stronger dissociation of this salt in the PEO matrix [110].

The free ion percentage is important in CPEs because the free ions contribute to ion conduction whereas ions pairs and ion aggregates do not. Research on this topic continues [116].

### 3.7. Effect of Nanoparticles on Transference Number of Composite Polymer Electrolytes

Following the discussion of polymer structure and interactions in CPEs, we proceed to address a parameter that is related to the ion conduction inside the polymer matrix. The transference number directly describes the charge transport and thus the current of a specific ion [117]. Specifically, the lithium ion transference number *t*^+^ indicates the fraction of the current carried by the cation (Li^+^) in the electrolytes. It is desirable to achieve a high *t*^+^ in order to enhance the electrode reaction kinetics and to eliminate the concentration gradients within the battery so that the internal voltage drop could be lowered and the output current increased [64]. The cation transference number is most commonly calculated by Equation (8) [118,119].

(8) $$t^{+}=\frac{\mu^{+}}{\mu^{+}+\mu^{-}}=\frac{D^{+}}{D^{+}+D^{-}}$$

In Equation (8), *t*^+^ is the transference number of cations. *D*^+^ and *D*^−^ stand for the cation and anion self-diffusion coefficients, μ^+^ and μ^−^ are the mobility [120,121] of the cation and anion, respectively.

High lithium transference number (*t*_Li_^+^) at ambient temperature contributes to efficient battery performance [122,123]. In view of the importance of *t*^+^, we discuss here two factors that influence *t*^+^: electrolyte state of matter (liquid, gel or solid) and nanoparticle surface properties (acidic/basic/neutral). 

The transference number of ions (*t*^+^ and *t*^−^) can be obtained from AC impedance spectroscopy [124,125], DC polarization electrochemical method [126,127], potentiometric measurements [128], and NMR [118,129,130,131]. A comparative study of these methods has been presented [132]. Table 4 gives *t*^+^ values for different systems [133]. In these samples, PEO (4 × 10^6^ g/mol) is denoted as 4mPEO and the organic solvents used were ethylene carbonate (EC) and diethyl carbonate (DEC), EO:Li = 10:1. The size of the nano-sized fumed silica added (content = 10 wt %) was not reported. 

**Figure 11:**
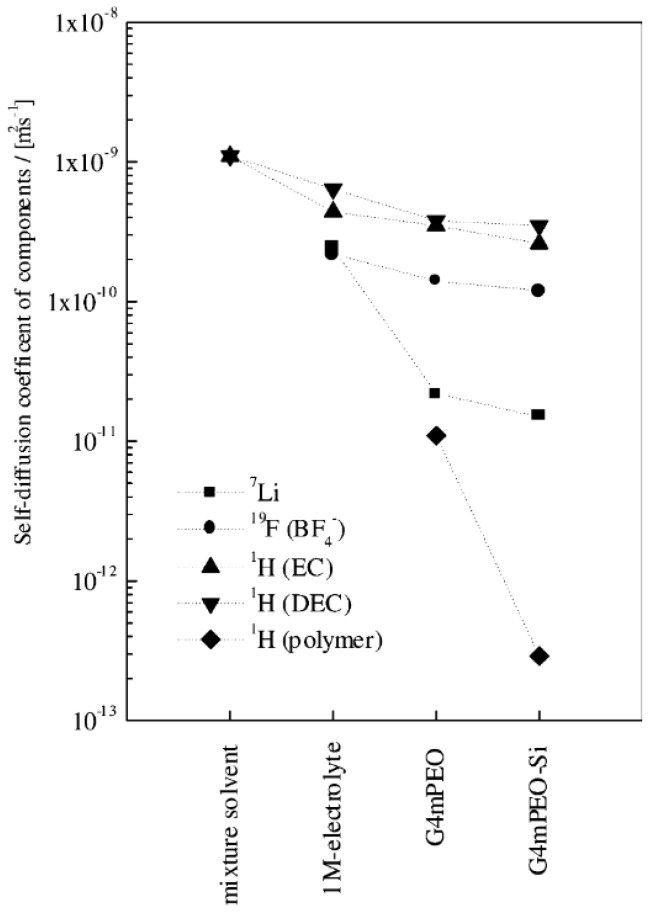
Self-diffusion coefficients of all diffusive components in ethylene carbonate-diethyl carbonate (EC–DEC) (0.27:0.75 mole fraction) mixed solvent, 1 M LiClO_4_ EC–DEC liquid electrolyte, swelled G4mPEO membrane and swelled composite G4mPEO–Si membrane (more information on the systems is provided in the text). Reproduced with permission from Aihara et al. [133]. Copyright 2002, Royal Society Chemistry.

We can see in Table 4 that the highest value of *t*^+^_NMR_ was 0.52 for the organic solvent-based liquid electrolyte of 1 M LiB_4_ in ethylene carbonate and diethyl carbonate mixture (EC–DEC), while in the Gel Polymer Electrolytes (GPE) *t*^+^ decreased to 0.14 and 0.11 for the system of G4mPEO (GPE with 4mPEO matrix swelled by EC-DEC), without and with 10 wt % fumed silica, respectively. Thus, the fumed silica decreased the *t*^+^. Also, the lower *t*^+^ matched the result [133] of lower conductivity for GPE at room temperature. Note that the *t*^+^_pol_ values obtained from electrochemical analysis were different from the *t*^+^_NMR_ from NMR, something that was ascribed to the motive forces of these two techniques [133]. Moreover, as shown in Figure 11, the larger values of the self-diffusion coefficients of the anion are higher than those of Li^+^, which indicates faster diffusion of the anion. This has been attributed to the interaction of Li^+^ with the polymer matrix [133].

The second factor affecting *t*^+^ is the active sites on the nanoparticle surface. For the system PEO–LiCF_3_SO_3_ (EO:Li^+^ = 20)–10 wt % Al_2_O_3_ (basic, neutral or acidic, *d* = 5.8 nm), the transference number *t*^+^ increased in the sequence of updoped (*t*^+^ = 0.46) < basic Al_2_O_3_ (*t*^+^ = 0.48) < neutral Al_2_O_3_ (*t*^+^ = 0.54) < acidic Al_2_O_3_ (*t*^+^ = 0.63) [134]. As for the explanation, the hydrogens of acidic ceramic surface (Lewis acid) form hydrogen bonds with the lithium salt anions and the ether oxygens (Lewis base) [134], which promote salt dissociation and also decrease the PEO crystallinity [123,134]. In this way, the transference number *t*^+^ increased. As for the neutral and basic Al_2_O_3_, the number of Lewis acid sites decreased leading to weaker increase in *t*^+^. This discussion would be more informative if the number of acidic sites on the surface could be quantified in combination with oxygen vacancy analysis. This would reveal the “efficiency of acidic site” on the *t*^+^ increase.

**Figure 12:**
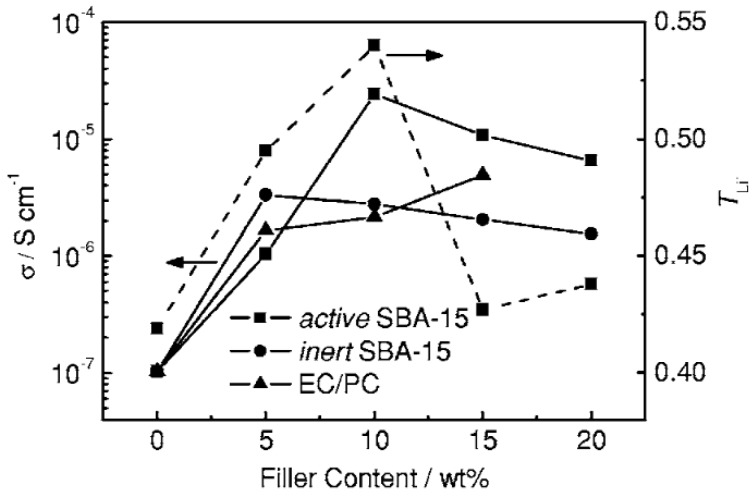
Ionic conductivity σ and lithium transference number *t*^+^ as a function of active SBA-15 (mesoporous silica) content for PEO (300,000 g/mol)–LiClO_4_–SiO_2_ (1000 m^2^∙g^−1^) CPE at 25 °C. For comparison, the ionic conductivity with inert SBA-15, as well as that with EC/propylene carbonate (PC), is also presented. Reproduced with permission from Wang et al. [64]. Copyright 2007, American Institute of Physics.

We discuss above factors affecting the lithium transference number *t*^+^. However, how is *t*^+^ related to conductivity? Conductivity and *t*_Li_^+^ results have been compared for CPEs, with the additive being ionically active/inert SiO_2_ particles (active SiO_2_ was mesoporous silica SBA-15 absorbing plasticizers of ethylene carbonate (EC)/propylene carbonate (PC); inert SiO_2_ was mesoporous silica SBA-15 without plasticizers) and organic solvent additive. As seen in Figure 12, for PEO (300,000 g/mol)–LiClO_4_ (EO:Li^+^ = 16)–SiO_2_ (1000 m^2^·g^−1^), the conductivity initially increased with addition of active SiO_2_, attained a maximum at 10 wt % active SBA-15 at a value of about 2.4 × 10^−5^ S∙cm^−1^, followed by a decline with further loading of active SBA-15 [64].

As a comparison, at the same optimum content of 10 wt % active SiO_2_, *t*_Li_^+^ reaches a maximum value of 0.54 [64]. This has been attributed to the competition of –OH on the surface of SBA-15 with Li^+^, both as Lewis acid, to coordinate with ether oxygens on the PEO chains and ClO_4_^−^ anions, both as Lewis base, which led to the promotion of desired Li^+^ transport by occupying more Lewis base coordination sites and thus *t*_Li_^+^ was enhanced. In the range of *t*_Li_^+^ increase with SBA-15 doping lower than 10 wt %, there are not enough –OH sites available for Lewis acid-base interaction. At content higher than 10 wt %, the trend of decreasing *t*_Li_^+^ could be ascribed to the aggregation of nanoparticles [64]. The simultaneous increase of *t*_Li_^+^ and conductivity from 0–10 wt % active SBA-15 loading indicates an effective way to increase conductivity by improving *t*_Li_^+^. The increase of both *t*_Li_^+^ and conductivity is required [135] for achieving high performance in lithium-ion batteries. 

## 4. Nanoparticle Additives Affect the Polymer Electrolyte Dielectric Properties

The dielectric properties of the polymer matrix are central to the understanding of the ion conducting behavior of polymer electrolytes. They are an important counterpart to polymer structure in determining the performance of CPEs as discussed in Chapter 3. However, the attention placed on this topic in the literature is much less than that on polymer structure. In this chapter, we review the effect of nanoparticles on dielectric properties of polymer electrolytes. 

### 4.1. Dielectric Constant (ε_r_ or ε’) and Dielectric Loss (ε_i_ or ε”)

The polymer dielectric constant and dielectric loss are of particular significance for the purpose of ion conduction [136]. On one hand, the dielectric constant determines the polymer’s ability to dissolve salts. High dielectric constant reduces ion-ion interactions and also inhibits crystal formation [137]. The dielectric constant of PEO below its melting temperature is reported in the range 3–4 [138], which is rated as a medium polarity. On the other hand, the dielectric loss helps to probe the relaxations, e.g., α relaxation [139,140] related to crystalline dipole rotation, β relaxation [141,142] related to dipole orientation in amorphous regions, and γ relaxation [143] related to the movement of side groups or end-groups in the amorphous phase. 

**Figure 13:**
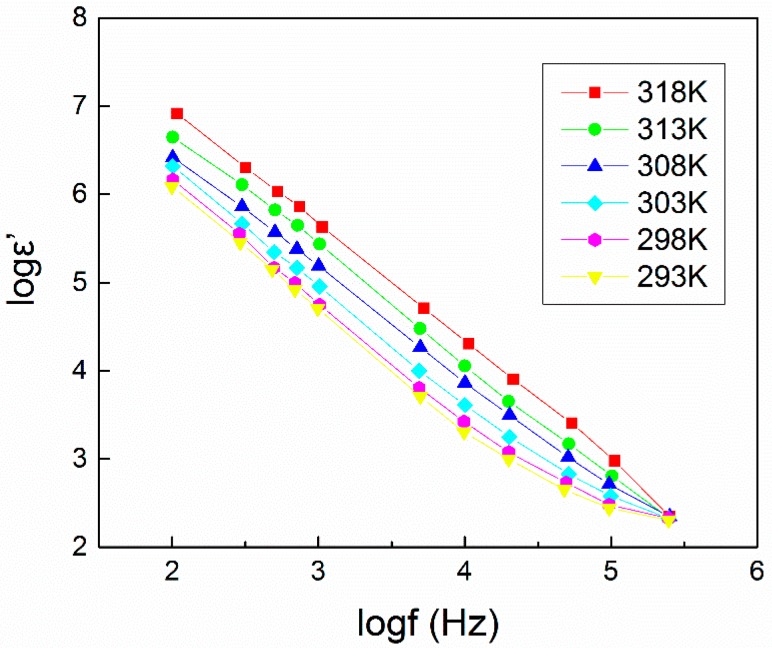
Variation of dielectric constant with frequency at different temperatures for PEO (8 × 10^6^ g/mol)–LiClO_4_ (EO:Li^+^ = 11)–LiAlO_2_ (*d* = 41 nm, *n*_nanoparticle_/*n*_Li_ = 1.5) CPE. Data from Masoud et al. [144].

A plot of dielectric constant (ε’) against frequency at different temperatures for PEO (8 × 10^6^ g/mol)–LiClO_4_ (EO:Li^+^ = 11)–LiAlO_2_ (d = 41 nm, *n*_nanoparticle_/*n*_Li_ = 1.5) is presented in Figure 13 [144]. The ε’ decrease with frequency increase can be ascribed to the lag of the dipole oscillating frequency compared to the frequency of the applied field [145]. At room temperature, the incorporation of LiAlO_2_ nanoparticles within the matrix increased the dielectric permittivity ε’ to 1.22 × 10^6^ F/m from the range of 9.8 × 10^3^–2.93 × 10^5^ F/m for additive-free samples [146]. An increasing temperature facilitates the dipole orientation and thus enhances the dielectric constant [147]. The temperature dependence of the dielectric constant at different frequencies can be roughly divided into two stages, a weak dependence at lower temperatures (293–303 K) due to the crystalline-to-amorphous transition [145], and a strong dependence at higher temperatures (303–318 K) due to a decrease of the CPE viscosity [147]. The frequency and temperature dependence of the dielectric loss (ε”) exhibit a similar trend with that of ε’ for different reasons [144]. The dielectric loss decrease with increasing frequency was attributed to high periodic reversal of the field, while the dielectric loss increase with temperature increase was due to the dipole relaxation and the decrease in the relaxation time [144]. Studies of ε’ and ε” in CPEs containing montmorillonite or carbon nanotubes (CNT) have been reported elsewhere [148,149].

### 4.2. Dielectric Relaxation Strength (Δε), Electrical Modulus (M) and Tangent Loss (tanδ)

Further analysis of ε’ and ε” can reveal insights into the dielectric behavior of CPEs and how this behavior affects CPE performance, e.g., ionic conductivity. After the dielectric constant (ε_r_) and dielectric loss (ε_i_) have been obtained, the electrical modulus *M*_r_ (real part) and *M*_i_ (loss part) can be introduced. The electric modulus spectra provide information about the ionic conduction relaxation process and they are calculated from the following equations [148]:

(9) $$M_{r}(\omega )=\frac{\varepsilon_{r}}{\left({\varepsilon_{r}}^{2}+{\varepsilon_{i}}^{2}\right)}$$

(10) $$M_{i}(\omega )=\frac{\varepsilon_{i}}{\left({\varepsilon_{r}}^{2}+{\varepsilon_{i}}^{2}\right)}$$

The tangent loss [148] is given by:

(11) $$\tan\delta =\frac{\varepsilon_{i}}{\varepsilon_{r}}=\frac{M_{i}}{M_{r}}$$

For example, these equations have been applied for the analysis of the system PEO (600,000 g/mol)–LiClO_4_ (EO:Li^+^ = 20)–*x* wt % MMT (Nano clay, Polymer Grade V, a product of Nanocor^®^) with varying amount of MMT [150]. The dielectric relaxation strength Δε measures the ionic polarization of the CPE. It equals the real part of dielectric constant at low frequencies ε’_s_ minus that at high frequency ε’_∞_, Δε = ε’_s_ − ε’_∞_ [151]. Δε can be estimated by using ε’ values at 1 kHz and 1 MHz. Δε = ε’ (1 kHz) − ε’ (1 MHz). At a fixed salt concentration of EO:Li^+^ = 20, the enhancement of Δε in Table 5 indicated an increase of ionic polarization as the MMT concentration increased [150]. As for the reason, it was speculated that the exfoliated MMT nanoplatelets helped reduce ion pairing, leading to a higher concentration of unpaired ions [150]. This result can be connected with the free ion percentage study highlighted in Section 3.6.

Besides the dielectric relaxation strength Δε for the understanding of ionic polarization, the polymer chain relaxation time reflects the mobility of polymer chains. Several relaxation times can be employed in order to describe the polymer chain relaxation behavior:

(12) $$\tau_{\varepsilon }=1/2\pi f_{p(\varepsilon_{i})}$$

(13) $$\tau_{\sigma }=1/2\pi f_{p(M_{i})}$$

(14) $$\tau_{\tan\delta }=1/2\pi f_{p(\tan\delta )}$$

**Figure 14:**
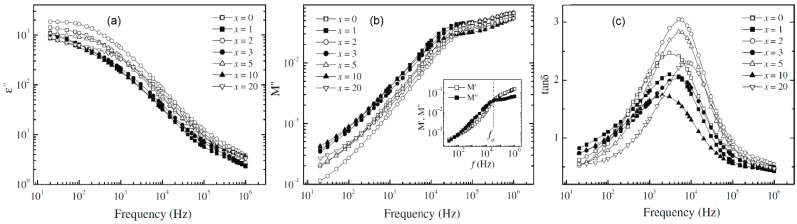
Frequency dependence of (**a**) dielectric loss ε”; (**b**) electric modulus loss *M*” and (**c**) loss tangent tanδ for CPEs of PEO (600,000 g/mol)–LiClO_4_ (EO:Li^+^ = 20)–*x* wt % MMT. Reproduced with permission from Choudhary [155]. Copyright 2013, National Institute of Science Communication and Information Resources (NISCAIR).

In the above equations, *f*_p_ stands for the frequency at the peak position in Figure 14. The subscripts: ε” (or ε_i_) is dielectric loss; tanδ is tangent loss; and *M*” (or *M*_i_) is loss part of electric modulus. Plots of ε”, tanδ and *M*” (or *M*_i_) against frequency are shown in Figure 14. The electric modulus (*M*) spectra can be employed to study the ionic conduction relaxation process of CPEs [152,153,154]. This ionic conduction relaxation frequency (*f*_σ_) is determined at the intersection of *M*’ and *M*” spectra as shown in the inset of Figure 14b [149] for CPE film of PEO (600,000 g/mol)–LiCF_3_SO_3_ (EO:Li^+^ = 20)–*x* wt % MMT. The ionic conduction relaxation time (τ_σ_) shown in column five in Table 5 was determined by τ_σ_ = (2π*f*_σ_)^−1^, wherein the ionic conductivity relaxation frequency (*f*_σ_) corresponds to a change in ion transport from DC to AC. 

From Table 5, we can see that the relaxation times (τ_ε”_, τ_tanδ_, and τ_M”_) decrease with an increase of MMT concentration, which confirms the increased segmental motion of Li^+^ cations that are coordinated with PEO polymer chains [150]. Such segmental motion facilitates the ion transport and thus results in an ionic conductivity improvement of the electrolytes.

### 4.3 AC/DC Conductivity and Impedance Spectroscopy

The conduction behavior of the CPEs under the condition of different AC frequency or DC is addressed in the section. The frequency dependence of the real part of AC conductivity (σ’) for PEO (600,000 g/mol)–LiCF_3_SO_3_/LiClO_4_ (EO:Li^+^ = 20)–*x* wt % MMT films has been reported [149,152]. The σ’ values of the MMT-filled CPEs were found higher than those without MMT, which was attributed to increased ion conduction paths resulting from the intercalated MMT structures [156,157]. All the σ’ spectra exhibit typically two linear slopes in the low and high frequency regions, respectively, due to the semi-crystalline PEO. At the low frequency range, a straight line fit of σ’ data was used to evaluate the DC ionic conductivity (σ_dc_) of these CPEs [149], as shown in column six of Table 5. The high frequency σ’ spectra of samples follow Jonscher’s power law [152] as shown in Equation (15).

(15) $$\sigma^{’}(\omega )=\sigma_{\mathrm{dc}}+A\omega^{n}$$

In this equation, *A* is a pre-exponential factor, *n* is a fractional exponent in the range 0–1 for ion conducting electrolytes, and ω = 2π*f* is the angular frequency.

The deviation of the σ_dc_ value in lower frequency (σ_dc_ decreases with frequency increase) is due to the electrode polarization (EP) effect [150], which occurs due to the formation of Electric Double Layers (EDLs). These layers are built up by the free charges at the electrolyte/electrode interface in plane geometry [151]. This EP effect causes an increase of the effective impedance between the electrodes, and thus decreases the AC conductivity. This electrode polarization relaxation frequency corresponds to the peak of the tanδ spectra [158,159,160] as shown in Figure 14c.

For the analysis of σ_dc_ change, we can also go back to its definition:

(16) $$\sigma_{dc}=\sum n_{i}\mu_{i}q_{i}$$

In this equation, *n*_i_ denotes the charge carrier density, μ_i_ the ion mobility and *q*_i_ is the charge of the *i* ion. Consequently, σ_dc_ can be attributed to (1) increased charge carrier density *n*_i_ by reduced ion-paring effect; and (2) increased ion mobility μ_i_ by increased polymer chain segmental dynamics.

**Figure 15:**
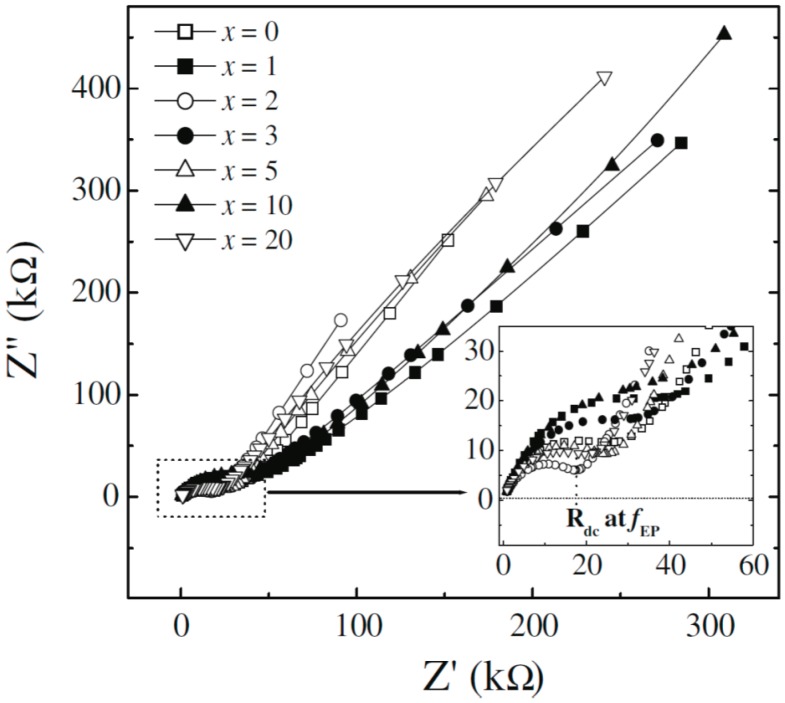
*Z*” versus *Z*’ plots of PEO–LiClO_4_ (EO:Li^+^ = 20)–*x* wt % MMT CPEs. The inset shows an enlarged view of the higher-frequency region. Reproduced with permission from Choudhary et al. [152]. Copyright 2011, National Institute of Science Communication and Information Resources.

The complex impedance plane plots of Z” against Z’ are shown in Figure 15 for PEO (600,000 g/mol)–LiClO_4_ (EO:Li^+^ = 20)–*x* wt % MMT systems, and the inset shows the zoom-in view of the plots at the high frequency range. (The frequency values increase from right to left in these plots.) The semicircular arcs in the high-frequency region correspond to properties of the bulk material; the spike in the lower-frequency region shows gradual increase of *Z*” values as the frequency decreases [149,150], which indicates a build-up of EDLs capacitance at the electrode/electrolyte interface. The DC impedance (*R*_dc_) is given by the extrapolated intercept on the real axis *Z*’ of the common part of two arcs. *R*_dc_ can then be used to estimate the σ_dc_ value by the following equation:

(17) $$\sigma_{\mathrm{dc}}=t/SR_{dc}$$

In Equation (17), *t* is the thickness of the electrolyte film and *S* is the effective area of the cell electrode. This estimated σ_dc_ value can be compared with the result from Jonscher’s power law (Equation (15)).

The impedance data (*Z*’ and *Z*”) can be related to dielectric properties (ε’ and ε”) by the following equations:

(18) $$\varepsilon^{’}=\frac{Z^{”}}{\omega C_{0}(Z^{’2}+Z^{”2})}$$

(19) $$\varepsilon^{”}=\frac{Z^{’}}{\omega C_{0}(Z^{’2}+Z^{”2})}$$

In these equations, *C*_0_ is the vacuum capacitance of the electrode, ε’ and ε” are real and imaginary permittivity respectively. Equations (18) and (19) connect the knowledge discussed in Section 4.3 to that of Section 4.1 and Section 4.2. 

In summary, analysis of dielectric constant (ε’) and dielectric loss (ε”) leads to electric modulus (*M*’ and *M*”), loss tangent (tanδ), and various relaxation times: τ_ε_, τ_tanδ_, τ_σ_, corresponding to the relaxation behaviors of coordinated polymer chains as given by Equations (11)–(14) [149,151,152,161,162,163]. Such analysis reveals two types of polarization: the ionic conduction polarization (also known as Maxwell-Wagner interfacial polarization) and the electrode polarization [151,162]. The ionic conductivity relaxation frequency (*f*_σ_) corresponding to change in ion transport from DC to AC can be determined at the intersection of *M*’ and *M*” spectra [149]. The electrode polarization relaxation frequency *f*_p(EP)_ is determined by the maximum in tanδ [151]. The Jonscher’s power law describes the frequency dependence of AC conductivity (σ’) at the high frequency range and estimates DC ionic conductivity (σ_dc_) at the low frequency range. Such dielectric studies (highlighted in Chapter 4) are as important for CPEs as the ion motion and polymer chain dynamics analysis (discussed in Chapter 3) [164,165]. 

## 5. Nanoparticle Additives Affect the Polymer Electrolyte Thermal and Mechanical Properties 

We discuss the effect of nanoparticles on polymer structure and interactions in Chapter 3 and on dielectric properties in Chapter 4 in order to provide fundamental explanations for the performance of CPEs, typically the ionic conductivity. With support of this knowledge we discuss in this chapter other elements of CPE performance, including mechanical and thermal properties, which are also important for the lithium-ion battery applications. 

### 5.1. Thermal Properties

Thermal properties, on one hand, reflect structure changes and, on the other hand, affect the performance of CPEs. For example, thermal parameters like the glass transition temperature (*T*_g_), melting temperature (*T*_m_), and melting enthalpy (Δ*H*) reflect polymer chain flexibility/rigidity and amorphous/crystalline percentage of the polymer. These thermal parameters also determine the operating temperature range of CPEs in lithium-ion batteries. 

For example, in a system where nano-sized CdO (d = 2.5 nm) was added into PEO (400,000 g/mol)–LiI electrolyte at a fixed ratio of EO/Li^+^ = 12 (CdO = 0.05–0.20 wt %), the values of *T*_g_, *T*_m_ and Δ*H* of PEO (400,000 g/mol)–LiI suddenly dropped with a small amount of added CdO as shown in Table 1 [63]. The lowest values were achieved simultaneously at 10 wt % CdO. Further addition of CdO caused less than 1 °C change for *T*_g_, while *T*_m_ and Δ*H* increased. The crystallinity (*χ* = Δ*H*/Δ*H*_PEO_, Δ*H*_PEO_ = 213.7 J/g [166]) decrease followed the same trend as that of Δ*H*, because the CdO nanoparticles lowered the reorganization tendency of PEO chains by the interaction with ether oxygens, leading to the formation of an amorphous interface region around the nanoparticles [167].

**Figure 16:**
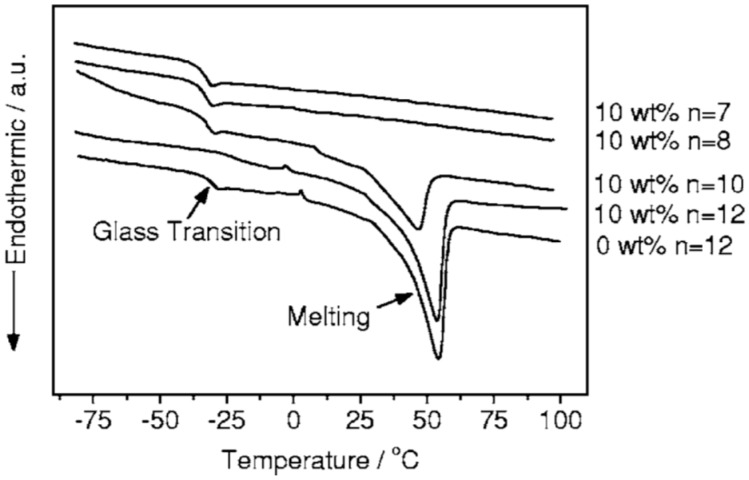
DSC of CPEs containing different wt % active SBA-15 and LiClO_4_ concentration (*n* = EO:Li^+^ molar ratio) as indicated in the legend. Reproduced with permission from Wang et al. [64]. Copyright 2007, American Institute of Physics.

Examples of the effect of lithium salt concentration on thermal properties have been reported [64]. At a fixed amount (10 wt %) of active SBA-15 (*A* = 1000 m^2^·g^−1^ mesoporous silica containing plasticizer) introduced into PEO (300,000 g/mol)–LiClO_4_ polymer electrolyte with EO:Li^+^ = 12, *T*_g_ increased from −30.4 °C for additive-free sample to −21.2 °C, and *T*_g_ dropped to a value −32 °C [64] when the concentration of lithium salts further increased (Figure 16). 

A report that nanoparticles had negligible effect on the *T*_g_ or crystallization of PEO [168] may be attributed to nanoparticle aggregation, resulting in only a small fraction of the nanoparticle surface being available to interact with the polymer chains. 

### 5.2. Mechanical Properties

Polymer electrolytes are promising to avoid drawbacks of the liquid state electrolytes and help to expand the operating conditions, even in harsh conditions, e.g., high temperature. However, the mechanical strength of pristine PEO is not satisfactory especially at a high working temperature due to its low melting temperature 66–75 °C. The binary systems with lithium salt do not exhibit an obvious improvement of mechanical strength despite the transient crosslinks [39] formed between lithium ions and ether oxygens. Thus, the mechanical properties of polymer electrolytes with nano-additives became a topic of research in order to further improve the mechanical strength of CPEs for batteries. In this section, we discuss how nanoparticles can improve mechanical properties such as tensile strength, yield strength, rheology, elastic and viscous modulus. 

#### 5.2.1. Tensile Strength and Yield Strength

**Figure 17:**
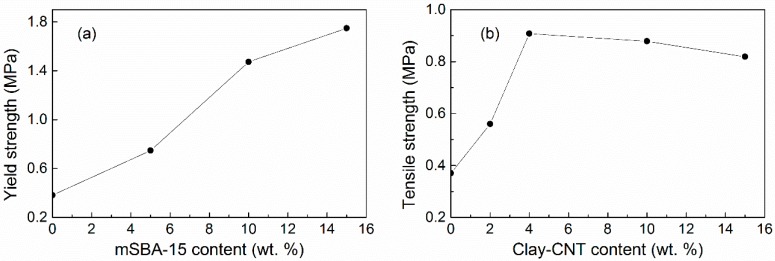
(**a**) Tensile stress–strain curves for PEO (300,000 g/mol)–LiClO_4_–mSBA (silane-functionalized mesoporous silica) CPE films with various amounts of mSBA-15; the inset shows their yield strength (room temperature). Data from Wang et al. [169]; (**b**) Tensile strength with varying hybrid filler content; the inset shows stress−strain curves of pristine PEO and PEO (100,000 g/mol)–LiClO_4_–(montmorillonite–CNT) samples with varying filler content. Data from Tang et al. [171].

The tensile modulus and yield strength of PEO (300,000 g/mol)–LiClO_4_–(mSBA-15: silane-functionalized mesoporous silica) increased with increasing amount of mSBA-15 in the 0–15 wt % range as shown in Figure 17a [169]. This enhancement is due to the added silica which acted as crosslinking media inside the polymer matrix by their surface interactions [170]. However, the enhancement was not always monotonic. For PEO (100,000 g/mol)–LiClO_4_–(montmorillonite–CNT), the optimized tensile strength was achieved upon 5 wt % filler incorporation as shown in Figure 17b. The result is 160% tensile strength increase compared to PEO electrolyte [171]. This reinforcement has been ascribed to the large aspect ratio and surface roughness of the clay–CNT hybrid filler, which lead to strong interactions with the polymer [171]. The different surface roughness may also explain the difference in the optimized nano-additive amount for maximized tensile strength between Figure 17a,b. In combination with the knowledge [63,64] that the maximum conductivity value occurred at 10 wt % nano-additive, the incorporation of 10 wt % nano-additive appears to offer an optimal combination of reinforced mechanical properties as well as improved ion conductivity. Of course, the surface conditions, e.g., roughness, functionalization, and surface defects may cause the final result to deviate. 

#### 5.2.2. Rheology, Elastic and Viscous Modulus

**Figure 18:**
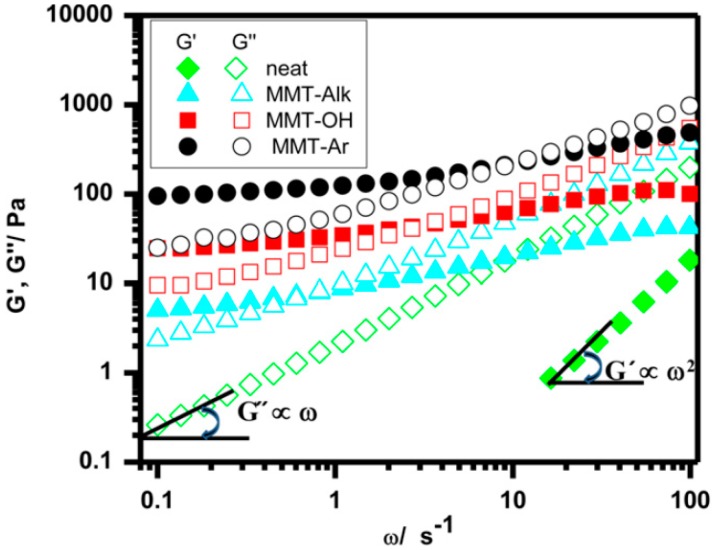
Frequency dependence of the storage (**filled symbols**) and loss (**open symbols**) modulus of PEO (8000 g/mol)–MMT nanocomposites at 75 °C. Reproduced with permission from Kelarakis et al. [174]. Copyright 2011, Elsevier Ltd., Amsterdam, The Netherlands.

As the mechanical strength is an important property of high molecular weight polymer-based CPEs for advanced lithium-ion batteries [172,173], for the low molecular weight counterpart CPEs, the corresponding rheological properties should be studied. The rheology of polymer composites (without lithium salt) is strongly influenced by the surface chemistry of the nanoparticles. For example, the rheology behavior of PEO (8000 g/mol) composite with different montmorillonites (MMT) (commercially available [174], particle size not reported) without lithium salt at 75 °C is shown in Figure 18 [174]. MMT with different substitutions of octadecyltrimethyl ammonium (MMT–Alk), hydroxide (MMT–OH) or aromatic groups (MMT–Ar) were employed for comparison. As shown in Figure 18, within the rheological window, the storage modulus of the composites was higher than that of the neat polymer. The storage modulus of hybrids increased in the sequence MMT–Alk < MMT–OH < MMT–Ar. The following reasons have been presented to explain this trend: for MMT–Alk additive, the weak interactions between the aliphatic chains of substitution and polymer chains made the rheology of the composite similar to that of the neat polymer; for the MMT–OH additive, the hydrogen bonding between the polar groups of substitution and the PEO ether oxygens made the elastic modulus increase; and for the MMT–Ar additive, charge transfer between benzene and ether oxygens exerted the greatest impact on the rheology of the composite, a result consistent with a more negative Flory-Huggins interaction parameter [175].

The similar trend of storage and loss modulus crossover frequency indicated the non-terminal relaxation mode of PEO (8000 g/mol)–MMT samples, and suggested the formation of interconnected network structure in the composites [176,177,178,179,180]. In future studies, such different functionalized MMT additives may also be applied to the analysis of nanoparticle effects on the viscosity and conductivity of CPEs with lithium salt present. 

In a similar study [181] for CPEs with oligomer PEO (*M*w ≈ 200 g/mol) matrix, rheology and conductivity data revealed no obvious change with the addition of different types of functionalized fumed silica (having silanol, alkyl, or PEO as surface groups). At high fumed silica content, the conductivity decreased only marginally [181]. It was speculated that for the case of a liquid oligomer matrix, the viscosity increase from nanoparticle doping impaired ion conduction. In contrast, for a solid polymer matrix, the surface functionalization of nanoparticle has been shown to be an effective way to simultaneously enhance conductivity and mechanical strength of CPEs [169,171]. Clearly, the nanoparticle effects differ depending on the liquid or solid state of the CPEs.

## 6. Processing Conditions Affect Composite Polymer Electrolyte Properties

Following the discussion on how the properties of composite polymer electrolytes are influenced by their constituent components, this section discusses the effects on the CPE performance of various experimental or processing conditions, including thermal history, aging, and humidity. All these conditions should be taken into consideration when the effects of other parameters are analyzed.

### 6.1. Thermal History

**Figure 19:**
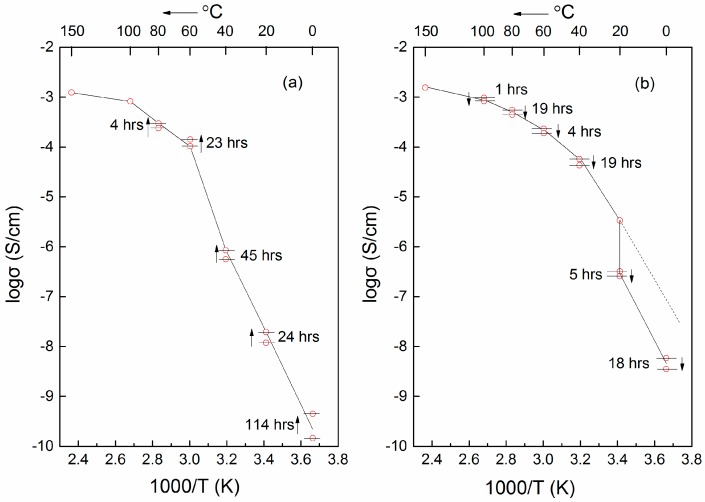
(**a**) Conductivity of PEO (2,000,000 g/mol)–LiBF_4_ (EO:Li^+^ = 8)–TiO_2_ (20 wt %, *d* = 21 nm) CPE heat-treated at 150 °C for 30 min and then quenched to 0 °C; (**b**) Conductivity of the same CPE heat-treated at 150 °C for 30 min. The conductivity was measured while the specimen was slowly cooled from the high temperature and then stabilized at the temperature of measurement. Data from Kumar et al. [182].

A typical study of the thermal history dependence concerns conductivity data for the system PEO (2,000,000 g/mol)–LiBF_4_ (EO:Li^+^ = 8)–TiO_2_ (20 wt %, with average *d* = 21 nm) [182,183]. Before the conductivity measurement, the sample was heated to 150 °C, equilibrated for 30 min and then quenched to 0 °C. During the heating process, the conductivity increased when the sample was held at different temperatures as shown in Figure 19a. This conductivity enhancement with holding time at different temperatures was attributed to dipole–dipole interactions [184]. At a high temperature of 150 °C, the thermal energy is sufficiently high to disrupt dipole–dipole interactions, and the TiO_2_ nanoparticles were in constant motion. Then the samples were cooled down slowly to temperature nodes of 100, 80, 60, 40, 20 and 0 °C for conductivity measurements [182,183]. The conductivity decreased marginally during the cooling process when the sample was held at a specific temperature above 20 °C. The dipole-dipole interactions were gradually restored, leading to the formation of charge double layer around the nanoparticles. However, a pronounced conductivity drop occurred at 20 °C, attributed to kinetic hindrance to PEO nucleation and crystallization in the presence of TiO_2_ particles [182]. It was also pointed out that (1) conductivity is higher in the cooling scan comparing to the heating scan from the comparison of Figure 19a,b; (2) Slow annealing during cooling scan helps to bring structural order in favor of ion conductivity [182]. Such a “structural order in favor of conductivity” appears to be a promising route for improving the overall performance of CPEs, as an alternative to tuning the CPE composition. 

### 6.2. Physical Aging

**Figure 20:**
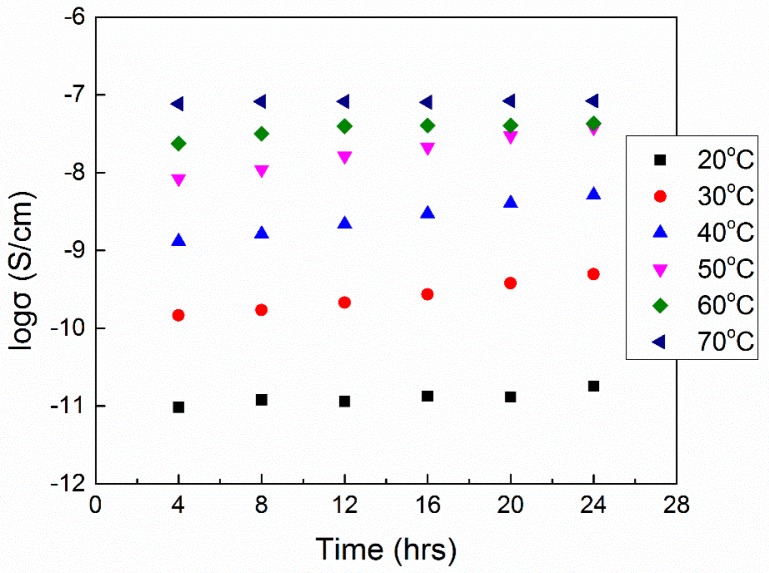
Time dependence of conductivity of the PEO–LiClO_4_ (EO:Li^+^ = 8:1)–Al_2_O_3_ (20 wt %, 24 nm) system at 20, 30, 40, 50, 60, and 70 °C. Data from Kumar et al. [183].

The second processing aspect to be discussed here is the aging effect on the composite polymer electrolyte conductivity by affecting the relaxation and crystallization. The conductivity relaxation of composite electrolytes at a given temperature can be expressed by Equation (20)

(20) $$\sigma^{’}(t)=\sigma (\infty )\pm \sum_{i=0}^{n}\sigma_{i}e^{-\nu_{i}t}$$

where σ’(*t*) represents the theoretical conductivity at time *t*; σ(∞) is the equilibrium conductivity; σ_i_ stands for the relaxation amplitude; *ν*_i_ is the relaxation frequency (1/τ_i_); and τ_i_ is the relaxation time. Equation (20) is simplified by assuming that only one type of relaxation mechanism is effective in the early stages of physical aging [182]:

(21) $$\sigma (t)=\sigma^{’}(t)-\sigma (\infty )=\sigma_{0}e^{-\nu_{0}t}$$

where σ(*t*) is the measured conductivity at time *t* [183]. The time dependence of conductivity for the amorphous sample PEO (2,000,000 g/mol)–LiClO_4_ (EO:Li^+^ = 8:1)–Al_2_O_3_ (20 wt %, 24 nm) at 20, 30, 40, 50, 60 and 70 °C is presented in Figure 20: the relaxation times τ_0_ obtained were 93, 36.6, 33.4, 31.45, 37 and 88.5 h, respectively. The lowest τ_0_ value of 31.45 h indicates that 50 °C is the temperature to facilitate fast structural stabilization. The conductivity relaxation was tracked only up to 24 h but physical aging may take months in order to reach an equilibrium [167].

For longer time scales, the time dependence of conductivity for PEO (600,000 g/mol)–LiClO_4_ (EO:Li = 8:1, 10:1)–Al_2_O_3_ (*d* = 4.8 ± 1.3 nm, concentration = 0, 5, 10, 25 wt %) [185], demonstrated the effect of crystallization on conductivity. Below the melting point (*T* < 65 °C), the conductivity decreased by several orders of magnitude because of the crystal structures formed after day 14. At temperatures above the melting point, the sample at day 1 and day 14 exhibited almost identical conductivity, even with different nanoparticle loading. It was thus noticed that for time scales within one day, the relaxation kinetics (relaxation time τ_0_) affected the conductivity, whereas for time scale longer than τ_0_, the crystallization of the sample dominated the conductivity change. Another conductivity relaxation study for the PEO (high molecular weight)–LiClO_4_ (EO:Li = 8)–10 wt % TiO_2_ (*d* = 13 nm) system at 31 °C showed an obvious drop between day 7 and day 8 due to the recrystallization kinetics that were speculated to be critically dependent on the annealing conditions [167].

### 6.3. Humidity

**Figure 21:**
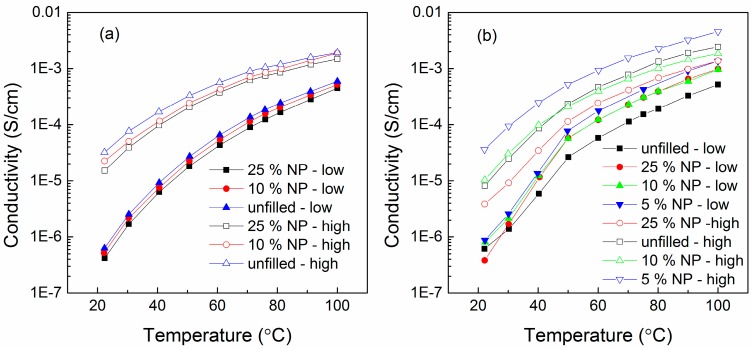
Conductivity vs. temperature at varying nanoparticle concentrations for samples held under conditions of high (**empty symbols**) and low (**filled symbols**) humidity at an EO:Li^+^ ratio of (**a**) 8:1 and (**b**) 10:1. Data from Fullerton-Shirey et al. [185].

Humidity is another experimental or processing factor to influence the CPE conductivity by disturbing the PEO crystallinity. Samples of PEO (600,000 g/mol)–LiClO_4_ (EO:Li = 8:1, 10:1)–Al_2_O_3_ (*d* = 4.8 ± 1.3 nm, amount = 0, 5, 10, 25 wt %) were exposed to air of 50% relative humidity for 3 days. As shown in Figure 21, conductivity increases were observed under humid conditions regardless of either nanoparticle concentration or temperature [185]. It was inferred that the weaker conductivity drop below the melting temperature *T*_m_ was due to the elimination of a small fraction of PEO crystallinity by the water present in humid samples [185].

(22) $$\gamma_{Water}(T)=\frac{\sigma_{X\%NP,Humid}}{\sigma_{X\%NP,Dry}}$$

(23) $$\gamma_{Water+NP}(T)=\frac{\sigma_{X\%NP,Humid}}{\sigma_{0\%NP,Dry}}$$

**Figure 22:**
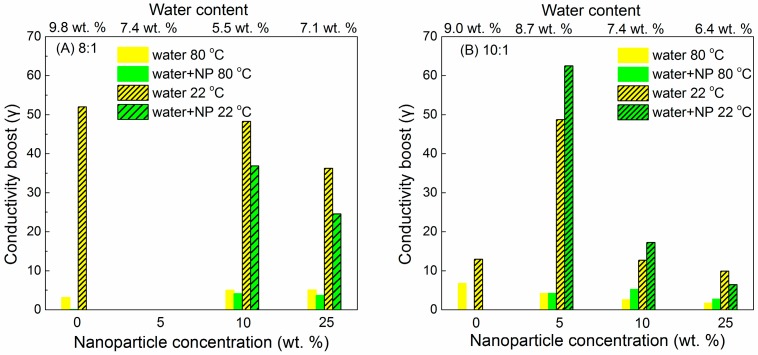
Conductivity boosts (“water boost”: γ_water_ and “water+NP boost”: γ_wate+NP_) at 80 and 22 °C at lithium concentrations of (**A**) 8:1 and (**B**) 10:1. The water fractions (wt %) above each nanoparticle concentration are the data for 22 °C. Data from Fullerton-Shirey et al. [185].

The conductivity increases caused by “water boost” (γ_water_) and “water + NP boost” (γ_water+NP_) are obtained by Equations (22) and (23). As shown in the example of Figure 22, when Al_2_O_3_ nanoparticles were added into the PEO (600,000 g/mol)–LiClO_4_ (EO:Li^+^ = 6) polymer electrolytes, (1) for all samples, the “conductivity boosts” below *T*_m_ (dashed lines) were significantly higher than those above *T*_m_ (solid lines); (2) when nanoparticles were introduced, the “conductivity boost” above *T*_m_ remained independent of the nanoparticle concentration while nanoparticles affected conductivity significantly below *T*_m_. These two phenomena have been explained by the dominant contribution of polymer chain mobility to conductivity above *T*_m_; (3) for EO:Li^+^ = 8:1 samples, the “water boost” was stronger than the “water + NP boost”, while for EO:Li^+^ = 10:1 samples, with 5 and 10 wt % nanoparticles, the “water boost” alone was less significant than that of “water + NP boost”; (4) the highest boost was achieved by the 10:1 sample as 5 wt %. The synergistic function of water and nanoparticles was described as follows: the nanoparticles preferentially aggregated in lithium-poor regions (pristine PEO) to allow more water in lithium-rich domains (PEO–LiClO_4_, EO:Li^+^ = 6) [185]. 

## 7. Summary and Outlook

Polymer electrolytes have attracted significant attention in electrochemical devices on the basis of their ability to overcome limitations of organic solvent-based electrolytes. Of particular interest are nanoparticles as additives to form composite polymer electrolytes (CPE) for applications in rechargeable batteries [22,23,24,26]. In this review, we focus on CPEs with conducting matrices based on PEO and conducting charge of Li^+^ from different lithium salts as shown in Table 6. We follow the basic principle that properties such as the structure and dielectric behavior of a CPE determine its performance in lithium-ion batteries. In order to explain the conductivity change of CPEs, we highlight studies that analyze each of the three components of the CPEs and their interactions, including (1) polymer matrix [58,59,62,63,65,66], with aspects of crystallinity, chain mobility and conformation [57,76,77,78,79,110], and dielectric properties [138,141,144,148,149,150,151,152,161,162]; (2) lithium salt, which deals with, e.g., cation transference number and free ion percentage; and (3) nanoparticle/salt ion/ether oxygen interactions. These are followed by a discussion of application-related thermal and mechanical properties of CPEs, and finally a presentation of processing conditions that may affect the CPE performance. 

In CPE research, the main objective of high ionic conductivity (10^−3^ S/cm at room temperature [135]) has rarely been reached, and an improvement of one performance aspect usually impairs another [186]. Generally, CPEs with 5–12 wt % nanoparticles (depending on additive type and surface activity, typically at 10 wt %) and molar ratio EO:Li^+^ = 6–20 (typically at 8–10) yield an optimal ionic conductivity. CPEs with optimized conductivity present lowest crystallinity (*χ*_c_), decreased glass transition temperature (*T*_g_) and melting temperature (*T*_m_). Meanwhile, the tensile strength can be improved several times compared to the undoped sample [63,64,133,167]. Of the processing factors discussed in Chapter 6, humidity is the most effective one to change the CPE ionic conductivity by more than one order of magnitude, this enhancement being more obvious at lower temperatures; thermal history affects the ionic conductivity close to an order of magnitude only for slowly cooled down samples at the room temperature range; the aging effect on conductivity is weak but more obvious in the temperature range 30–50 °C. These thermal and mechanical properties, together with processing factors should all be taken into consideration in the context of CPE performance in practical applications. 

Although the CPEs under investigation are experiencing a bottleneck in that the highest ionic conductivity reported for solid state CPEs just got close to 10^−3^ S/cm at room temperatures [187,188,189], this topic is still promising. Future research in this field may fall into two broad directions. The first direction is the easier and more practical way to adjust the CPE composition, with incorporation in the CPE of certain other additives beyond the three basic ingredients of polymer matrix, lithium salt and nanoparticle [64,190,191,192]. The second direction encompasses three-dimensional nano-level structural organization [1,2,3,171], such as the formation of ion tunnels [60] or ion paths [193], to facilitate ion conduction. Polymer matrix modification by side chain grafting [194,195] or attaching a different-type block [196,197,198] on the polymer backbone can also be viewed as part of this direction if it enables the formation of ordered structure. Only a few researches [171,189,199] have followed this second direction. The knowledge network presented in this review can be applied for the study of newly designed CPEs.

## Figures and Tables

**Table 1:** Comparison of thermal parameters obtained from differential scanning calorimetry (glass transition temperature, *T*_g_, melting temperature *T*_m_, enthalpy change Δ*H*, and fraction of crystalline region in the polymer referred to as crystallinity *χ*_c_) of PEO-LiI-CdO composite polymer electrolytes (CPEs). Adapted from Karmakar et al. [63].

CdO (wt %)	*T*_g_ (°C) (±2)	*T*_m_ (°C) (±2)	Δ*H* (J·g^−1^)	*χ*_c_ (%)
0	−36.4	55.9	51.9	24.3
0.05	−49.6	46.8	28.8	13.4
0.10	−48.9	45.1	25.3	11.8
0.15	−49.9	51.2	39.3	18.4
0.20	−49.6	53.2	41.0	19.2

**Table 2:** Trends for *T*_g_^NMR^, *T*_max_, and Δ*ν* with an increase of the *X*, *n*, and *Y* parameters of Types I and II ormolytes (hybrid organic-inorganic ionic conductors). Adapted from Mello et al. [76].

	Series 1	Series 2	Series 3	Series 4
Composition	[41]_6_[4]-I	[58]_6_[4]-I	[58]_6_[4]-I	[76]_17_[4]-II
	[58]_6_[4]-I	[58]_12_[4]-I	[58]_6_[8]-I	[76]_17_[8]-II
	[73]_6_[4]-I	[58]_20_[4]-I	[58]_6_[10]-I	[76]_17_[10]-II
	[78]_6_[4]-I		[58]_6_[15]-I	[76]_17_[15]-II
	[83]_6_[4]-I		[58]_6_[30]-I	[76]_17_[30]-II
	[91]_6_[4]-I		[58]_6_[80]-I	
	[95]_6_[4]-I			
*T*_g_^NMR^ (°C)	Approximately constant *X* < 80: −32 ± 5 *X* > 80: −48 ± 5	Increase −37 → −28	Approximately constant −38 ± 5	Decrease 7 → −16
*T*_max_ (°C)	Approximately constant *X* < 80: 30 ± 5 *X* > 80: 21 ± 5	Increase 26 → 46	Approximately constant 19 ± 5	Decrease 81 → 43
Δ*υ* (kHz)	Increase 5.4 → 8.0	Approximately constant 6.4 ± 0.3	Decrease 6.4 → 2.7	Increase 5.7 → 6.7

**Table 3:** Structural and adsorbed oxygen percentage of Sn atoms in SnO_2_ nanoparticles within the system PEO (600,000 g/mol)–LiClO_4_ (EO:Li^+^ = 8)–SnO_2_ (*d* = 3–4 nm, weight ratio of SnO_2_:PEO = 0.05, 0.10, 0.15, 0.20). Adapted from Xiong et al. [105]. Samples B, C and D were obtained by calcining sample A (synthesized SnO_2_) at 400, 600 and 800 °C for 3 h.

Sample	A	B	C	D
Structural O atoms	86%	87%	89%	83%
Adsorbed O atoms	45%	39%	36%	44%

**Table 4:** Transference number of gel electrolyte membranes. Adapted from Aihara et al. [133].

Sample	*t*^+^_NMR_^a^	*t*^+^_pol_^b^
1M LiBF_4_ in EC–DEC	0.52	-
G4mPEO swelled by EC–DEC	0.14	0.34
G4mPEO–10 wt % SiO_2_, swelled by EC–DEC	0.11	0.40

^a^ *t*^+^_NMR_ = values determined by NMR; ^b^ *t*^+^_pol_ = valued determined by electrochemical technique.

**Table 5:** Values of real part of permittivity ε’ at 1 kHz and 1 MHz, dielectric relaxation strength Δε = ε’ (1 kHz) − ε’ (1 MHz), dielectric relaxation time τ_ε_, loss tangent relaxation time τ_tanδ_, conductivity relaxation time τ_σ_, and DC ionic conductivity σ_dc_ of ball mill blended solution-cast electrolyte films PEO–LiClO_4_ (EO:Li^+^ = 20)–*x* wt % MMT. Adapted from Choudhary [150]. Copyright, 2013, National Institute of Science Communication and Information Resources (NISCAIR).

*x* wt % MMT	Δε	τ_ε_ (μs)	τ_tanδ_ (μs)	τ_σ_ (μs)	σ_dc_ × 10^7^ (S/cm)
0	18.39	17.11	7.58	3.46	0.54
1	21.19	11.58	5.35	2.24	0.66
2	29.14	7.99	3.42	1.47	1.62
3	29.45	9.53	4.00	1.64	1.60
5	94.59	9.29	2.71	0.10	3.91

**Table 6:** Chemical structures of lithium salts and organic solvents mentioned in this review.

**Lithium trifluoromethanesulfonate, lithium triflate (CF_3_SO_3_Li)**	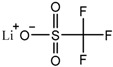
**Lithium bis(trifluoromethanesulfonyl) imide [LiN(SO_2_CF_3_)_2_]**	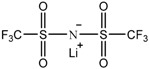
**Lithium tetrafluoroborate (LiBF_4_)**	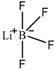
**Lithium hexafluorophosphate (LiPF_6_)**	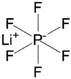
**Lithium perchlorate (LiClO_4_)**	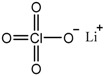
**Lithium iodide (LiI)**	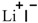
**Ethylene carbonate (EC),** **(CH_2_O)_2_CO**	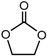
**Propylene carbonate (PC), CH_3_C_2_H_3_O_2_CO**	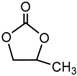
**Diethyl carbonate (EDC), OC(OCH_2_CH_3_)_2_**